# Factors associated with immune responses to SARS-CoV-2 vaccination in individuals with autoimmune diseases

**DOI:** 10.1172/jci.insight.180750

**Published:** 2024-06-04

**Authors:** Erik Anderson, Michael Powell, Emily Yang, Ananya Kar, Tung Ming Leung, Cristina Sison, Rebecca Steinberg, Raven Mims, Ananya Choudhury, Carlo Espinosa, Joshua Zelmanovich, Nkemakonam C. Okoye, Eun Jung Choi, Galina Marder, Sonali Narain, Peter K. Gregersen, Meggan Mackay, Betty Diamond, Todd Levy, Theodoros P. Zanos, Arezou Khosroshahi, Ignacio Sanz, Eline T. Luning Prak, Amit Bar-Or, Joan Merrill, Cristina Arriens, Gabriel Pardo, Joel Guthridge, Judith James, Aimee Payne, Paul J. Utz, Jeremy M. Boss, Cynthia Aranow, Anne Davidson

**Affiliations:** 1 Institute of Molecular Medicine, Feinstein Institutes for Medical Research, Northwell, Manhasset, New York, USA.; 2 Donald and Barbara Zucker School of Medicine at Hofstra/Northwell, Hempstead, New York, USA.; 3 Department of Microbiology and Immunology, School of Medicine, Emory University, Atlanta, Georgia, USA.; 4 Division of Immunology and Rheumatology, Department of Medicine, and; 5 Institute for Immunity, Transplantation and Infection, School of Medicine, Stanford University, Stanford, California, USA.; 6 Biostatistics Unit, Office of Academic Affairs, Northwell, New Hyde Park, New York, USA.; 7 Institute of Bioelectronic Medicine, Feinstein Institutes for Medical Research, Northwell, Manhasset, New York, USA.; 8 Department of Pathology and Laboratory Medicine, Donald and Barbara Zucker School of Medicine at Hofstra/Northwell, Hempstead, New York, USA.; 9 Department of Dermatology, Vagelos College of Physicians and Surgeons, Columbia University, New York, New York, USA.; 10 Division of Rheumatology, Department of Medicine, School of Medicine, Emory University, Atlanta, Georgia, USA.; 11 Department of Pathology and Laboratory Medicine, and; 12 Department of Neurology, Perelman School of Medicine, University of Pennsylvania, Philadelphia, Pennsylvania, USA.; 13 Oklahoma Medical Research Foundation and University of Oklahoma Health Sciences Center, Oklahoma City, Oklahoma, USA.

**Keywords:** Autoimmunity, Autoimmune diseases

## Abstract

Patients with autoimmune diseases are at higher risk for severe infection due to their underlying disease and immunosuppressive treatments. In this real-world observational study of 463 patients with autoimmune diseases, we examined risk factors for poor B and T cell responses to SARS-CoV-2 vaccination. We show a high frequency of inadequate anti–spike IgG responses to vaccination and boosting in the autoimmune population but minimal suppression of T cell responses. Low IgG responses in B cell–depleted patients with multiple sclerosis (MS) were associated with higher CD8 T cell responses. By contrast, patients taking mycophenolate mofetil (MMF) exhibited concordant suppression of B and T cell responses. Treatments with highest risk for low anti–spike IgG response included B cell depletion within the last year, fingolimod, and combination treatment with MMF and belimumab. Our data show that the mRNA-1273 (Moderna) vaccine is the most effective vaccine in the autoimmune population. There was minimal induction of either disease flares or autoantibodies by vaccination and no significant effect of preexisting anti–type I IFN antibodies on either vaccine response or breakthrough infections. The low frequency of breakthrough infections and lack of SARS-CoV-2–related deaths suggest that T cell immunity contributes to protection in autoimmune disease.

## Abstract 2

Individuals with an autoimmune disease have low IgG responses but normal or enhanced T cell responses to SARS-CoV-2 vaccine with minimal induction of disease flares or autoantibodies and low frequency of breakthrough infections.

## Introduction

Vaccinating individuals with an autoimmune disease is imperative in healthcare due to these individuals’ heightened susceptibility to severe infections, including SARS-CoV-2 (1). Understanding vaccine responses in patients with autoimmune diseases is crucial, as they may face increased risk of adverse reactions and mount less efficient immune responses (2). Many vaccines contain immune adjuvants that could worsen existing autoimmunity (2). Of particular concern for patients with systemic lupus erythematosus (SLE) is the fact that most SARS-CoV-2 vaccines contain mRNA that could act as a TLR7/8 agonist (3). Additionally, medications used in autoimmune populations have been linked to reduced antibody responses to SARS-CoV-2 vaccination (4–10). Strategies like withholding immunosuppressive medications prior to vaccination result in higher rates of seroprotection (11–13). Some studies have explored the effect of immunosuppression on T cell effector responses to SARS-CoV-2 vaccines during conventional or biologic immunotherapies (6, 14–17). While T cell immunity alone does not prevent SARS-CoV-2 infection, evidence suggests it limits disease severity (18, 19). Recent studies also suggest that antibodies to type I IFN and other cytokines may negatively affect responses to infections and vaccinations, although the effects are still debated (20–22).

The COVID-19 pandemic provided an opportunity to examine how autoimmune diseases and immunosuppressive medications influence responses to new lipid nanoparticle–encased mRNA vaccines. In this real-world observational study, we assessed factors affecting B and T cell vaccine responses in patients with an autoimmune disease and evaluated the association of vaccination with disease activity, frequency of breakthrough infections, association of infections with anti–type I IFN antibodies, and induction of autoantibodies following vaccination and boosting. The results of our studies help inform best practices for performing vaccination and boosting in patients with a broad spectrum of autoimmune diseases and those who are being treated with a range of immunotherapies.

## Results

### Patient characteristics.

**Figure 1:**
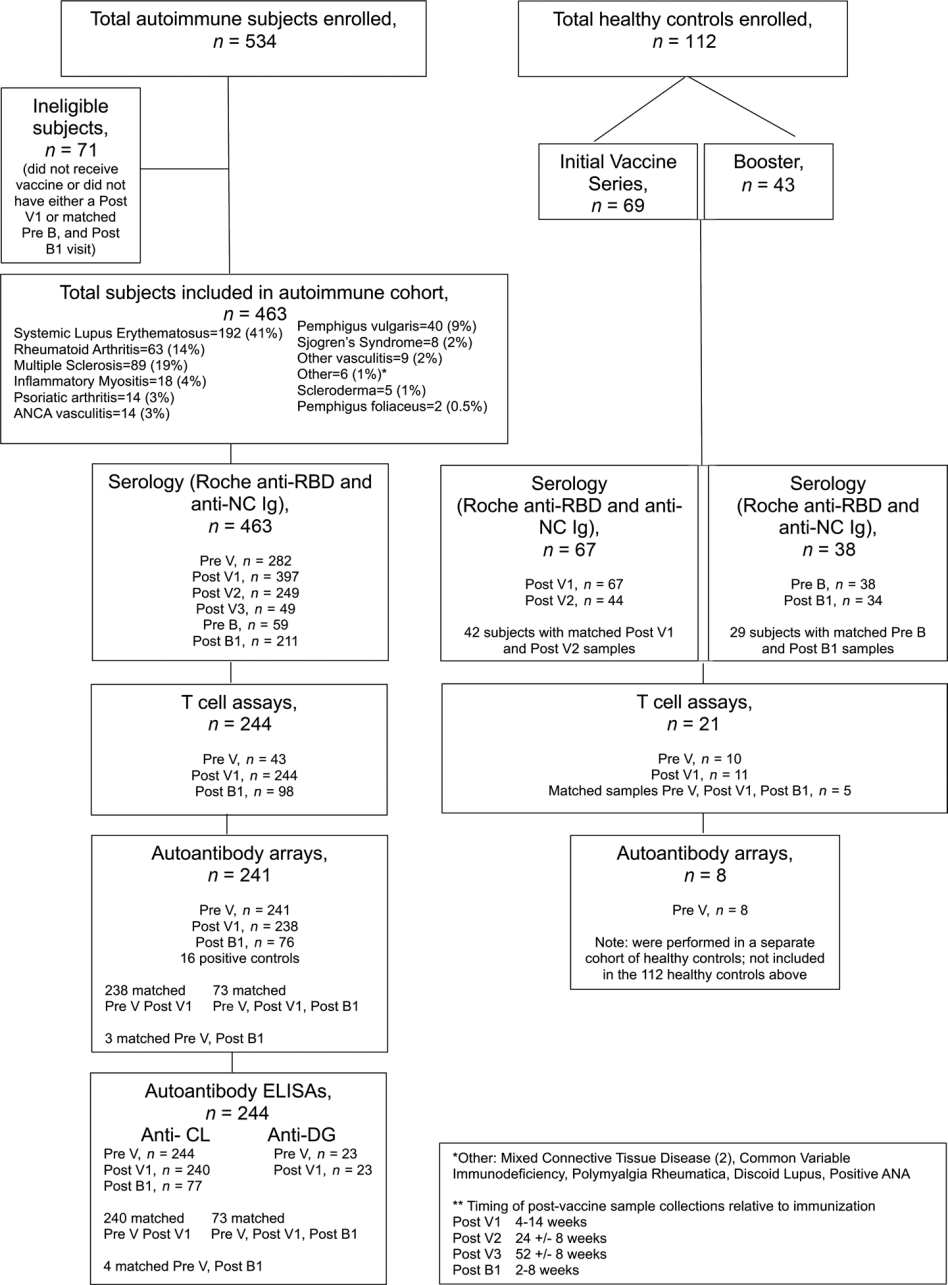
Patient recruitment. Flow chart of patients and analyses. Pre V, Prevaccine visit; Post V1, first visit 4–14 weeks after completion of first vaccine series; Post V2, second visit 24 ± 8 weeks after completion of first vaccine series; Post V3, third visit 52 ± 8 weeks after completion of first vaccine series; Pre-B, day of first booster; Post B1, visit 2–8 weeks after first booster.

Five hundred and thirty-four patients consented to the study. Seventy-one patients were subsequently deemed ineligible because they were not vaccinated or did not have a follow-up visit within the required period after vaccination. Demographic data are shown in Table 1, and distribution of autoimmune diseases and number of patients analyzed in each assay are shown in Figure 1.

### Serologic response to SARS-CoV-2 vaccine in patients with autoimmune diseases versus healthy controls.

**Figure 2:**
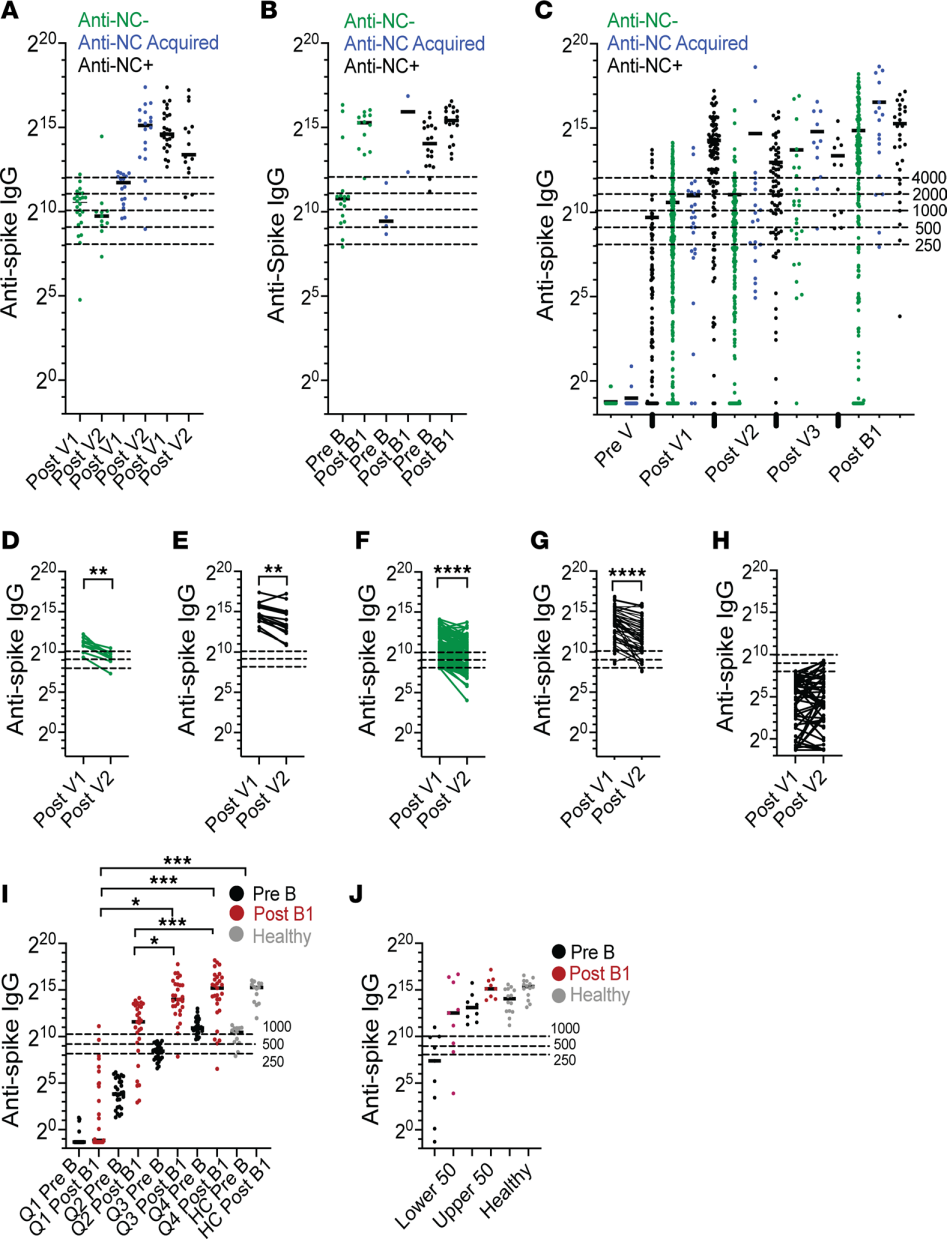
Serological response to SARS-CoV-2 vaccination in patients with autoimmune diseases versus healthy controls according to SARS-CoV-2 exposure status. Patients are divided into anti-nucleocapsid (anti-NC) IgG^–^ (green, no SARS-CoV-2 infection documented throughout the study), anti–NC Acquired (anti–NC Acq) (blue, SARS-CoV-2 infection documented at or after Post V2), and anti-NC^+^ (black, SARS-CoV-2 infection documented before initial vaccination) groups. (**A** and **B**) Anti–spike IgG levels (U/mL) in HC at each visit after the initial (**A**) and booster vaccination (**B**). (**C**) Anti–spike IgG levels in patients with autoimmune diseases at each visit before and after the initial vaccination and after booster vaccination. Statistical analyses are shown in Supplemental Figure 2. (**D** and **E**) Trajectory of anti–spike IgG levels after the initial vaccine series in anti-NC^–^ (**D**) and anti-NC^+^ (**E**) HC who responded to the initial vaccination. (**F** and **G**) Trajectory of anti–spike IgG levels after the initial vaccine series in anti-NC^–^ (**F**) and anti-NC^+^ (**G**) patients with autoimmune diseases who responded to the initial vaccination. (**H**) Trajectory of anti–spike IgG levels after the initial vaccine series in anti-NC^–^ patients with autoimmune diseases with an inadequate response (<250 U/mL). (**I**) Anti–spike IgG levels in anti-NC^–^ patients with autoimmune diseases versus HC before and after booster vaccination according to quartile of prebooster anti–spike IgG levels. (**J**) Anti–spike IgG levels in anti-NC^+^ patients with autoimmune diseases versus HC before and after booster vaccination according to the upper and lower 50th percentile of prebooster anti–spike IgG levels. Each data point represents an individual patient. (**D**–**G**) Wilcoxon signed-rank test. (**I** and **J**) Kruskal-Wallis ANOVA with DSCF correction for multiple comparisons. *P < 0.05, **P < 0.01, ***P < 0.001, ****P < 0.0001. Timing of sample collections is shown in Figure 1.

We assessed anti-nucleocapsid (anti-NC) and anti–spike IgG antibody values and trajectories in healthy control (HC) and autoimmune cohorts (Supplemental Figure 1, A–C; supplemental material available online with this article; https://doi.org/10.1172/jci.insight.180750DS1). Anti–spike IgG values showed no correlation with age or sex in patients with autoimmune diseases (Supplemental Figure 1D). To assess the effect of previous SARS-CoV-2 infection on vaccine responses, patients and controls were divided into 3 groups based on their prior exposure to SARS-CoV-2: (a) those who remained anti-NC^–^ with no symptomatic infection (anti-NC^–^); (b) those who, at the prevaccine (Pre V) visit, were positive for anti-NC or anti–spike IgG antibodies, or for SARS-CoV-2 by PCR or antigen test, or who at the first postvaccine (Post V1) visit 4–14 weeks after completion of initial vaccine, were anti-NC^+^ or reported a symptomatic infection that predated vaccination verified by positive PCR or antigen test (anti-NC^+^); and (c) those acquiring anti-NC positivity or testing positive for SARS-CoV-2 at or after the second postvaccine (Post V2) visit 24 ± 8 weeks after completion of the initial vaccination series (anti–NC Acq). Twenty-four patients with positive anti-NC antibodies at the Post V1 visit but no history of symptomatic infection were considered to be anti-NC^+^. Since cutoffs for anti–spike IgG positivity are not clearly defined, we based cutoffs on anti–spike IgG responses at each visit in HC (Figure 2, A and B). We defined a low anti–spike IgG response to the initial vaccine series as < 250 U/mL for anti-NC^–^ patients and < 2,000 U/mL for anti-NC^+^ patients and a low anti–spike IgG response to the booster vaccine as < 4,000 U/mL in all patients. These cutoffs are similar to those recently reported in a similar study from the United Kingdom (23).

Anti–spike IgG values in patients with autoimmune diseases at the Pre V visit are shown in Supplemental Figure 1, E–H. Anti-NC^+^ patients had significantly higher anti–spike IgG values at Post V1 than both anti-NC^–^ and anti–NC Acq patients (Figure 2, A and C, Supplemental Figure 1, F and I, and Supplemental Table 1). In total, 45.1% of anti-NC^–^ patients with autoimmune diseases had a serologic response of < 250 U/mL at Post V1, while only 1 of 24 (1.5%) of HC (an 83-year-old male) had a low response. Similarly, 25.5% of anti-NC^+^ patients with autoimmune diseases had a serologic response of < 2,000 U/mL at Post V1, compared with 0 of 27 HC. At the postbooster (Post B1) visit, 42.1% of anti-NC^–^ and 27.7% of anti-NC^+^ patients with autoimmune diseases had a serologic response of < 4,000 U/mL, compared with none of the HC (Figure 2, B and C; Supplemental Figure 1, H and I; and Supplemental Table 1). The percent increase in the anti–spike IgG antibody response at the Post B1 compared with the Post V1 visit was lower in anti-NC^+^ HC who already had high values of anti–spike IgG, compared with anti-NC^–^ HC (median fold increase 3.7 vs. 21.2, *P* < 0.0001; Supplemental Figure 1J); however, it was not different in patients with autoimmune diseases (Supplemental Figure 1, K and L; median fold increase 17.4 versus 11.0).

We next examined the decline in anti–spike IgG values after the initial vaccine series. The decline in anti–spike IgG between Post V1 and Post V2 in either anti-NC^–^ or anti-NC^+^ patients with autoimmune diseases was no different than in matched HC (Figure 2, D–G). Few nonboosted patients with autoimmune diseases could be observed at Post V3, as most had either received booster vaccinations or had acquired SARS-CoV-2 after Post V2. IgG anti–spike IgG values continued to decline in these patients (Supplemental Figure 1M). In anti-NC^–^ patients with autoimmune diseases who had a response < 250 U/mL to the initial vaccine series, anti–spike IgG remained stable from Post V1 to Post V2 (Figure 2H).

To determine whether the anti–spike IgG response to booster vaccination was predicted by the prebooster anti–spike IgG level, we compared booster vaccination responses based on prebooster (Pre B) anti–spike IgG quartiles. Anti-NC^–^ patients with autoimmune diseases in the highest 2 quartiles at the Pre B visit had significantly higher anti–spike IgG at Post B1 (equivalent to HC), unlike those in the lower 2 quartiles (Figure 2I). By contrast, no difference was observed in anti–spike IgG between the upper and lower 50th percentile of Pre B levels in anti-NC^+^ patients with autoimmune diseases (Figure 2J).

### Immunosuppressant use and serologic response to vaccine.

We assessed the effect of immunosuppressant drugs on the response to vaccination in 397 patients with a Post V1 visit (Supplemental Table 2). Only 32% of 96 patients on B cell–depleting drugs were responders. Previous exposure to SARS-CoV-2 did not improve vaccine responses in this cohort. To evaluate the effect of mycophenolic acid (MPA) and mycophenolate mofetil (MMF) on the vaccine response, we excluded patients also taking B cell–depleting drugs and those who were nonadherent to their medication based on serum drug levels. Sixty-one percent of 49 patients taking MPA or MMF alone or together with hydroxychloroquine were responders. In this cohort, all 14 anti-NC^+^ patients were responders, compared with only 46% of 35 anti-NC^–^ patients (*P* < 0.01). Only 26% of 19 patients taking MPA or MMF together with a second immunosuppressive drug were responders (*P* < 0.01, compared with patients taking MPA or MMF alone or together with hydroxychloroquine). Seventy-three percent of 49 patients taking methotrexate and 68% of 16 patients taking azathioprine were responders, and all nonresponders were anti-NC^–^. Similarly, 73% of 26 patients taking TNF inhibitors were responders and 4 of 5 nonresponders were anti-NC^–^. Finally, only 9 of 25 (36%) patients taking belimumab were responders; these 9 patients were taking belimumab alone (1 of 9) or with MMF (1 of 9), methotrexate (2 of 9) or hydroxychloroquine (5 of 9). Of the belimumab NR, 11 of 16 were also taking other immunosuppressives including MMF (9 of 11), azathioprine (1 of 11), or methotrexate (1 of 11), and all were anti-NC^–^. Thus, the combination of MMF and belimumab conferred a 90% risk of NR.

Among 153 patients with both a Post V1 and Post B1 visit, we assessed medication use among those who did not respond to both the initial vaccine series and the booster (“Double Non-Responders” [Double NR], *n* = 36), those who did not respond to the initial vaccine series but responded to the booster (“Single Non-Responders” [Single NR], *n* = 41), and those who responded to both vaccinations (“Responders,” *n* = 76; Supplemental Table 3). A majority of Double NR received B cell depletion alone (20 of 36, 56%) or with another drug (6 of 36, 9%). The other Double NR patients received belimumab with a second immunosuppressive drug (4 of 36), MMF alone (1 of 36), and leflunomide together with a TNF inhibitor (1 of 36). In addition, 4 of 5 patients receiving fingolimod were Double NR, while the other was a Single NR. Most patients who were on MMF alone or with hydroxychloroquine were Responders (8 of 13, 63%). However, all patients who were on MMF with an additional immunosuppressant (*n* = 8) were either Single NR (5 of 8) or Double NR (3 of 8). Ten of 13 (77%) patients on belimumab were either Single NR (6 of 10) or Double NR (4 of 10). Of 16 patients on methotrexate, 50% were Single NR and 50% were Responders, while none were Double NR. Only 5 patients were taking a prednisone equivalent of 30 mg daily at the time of the initial vaccine series, and only 1 patient was taking this dose of corticosteroid at the time of the booster.

### Vaccine type, immunosuppressant adjustment, and vaccine response.

**Figure 3:**
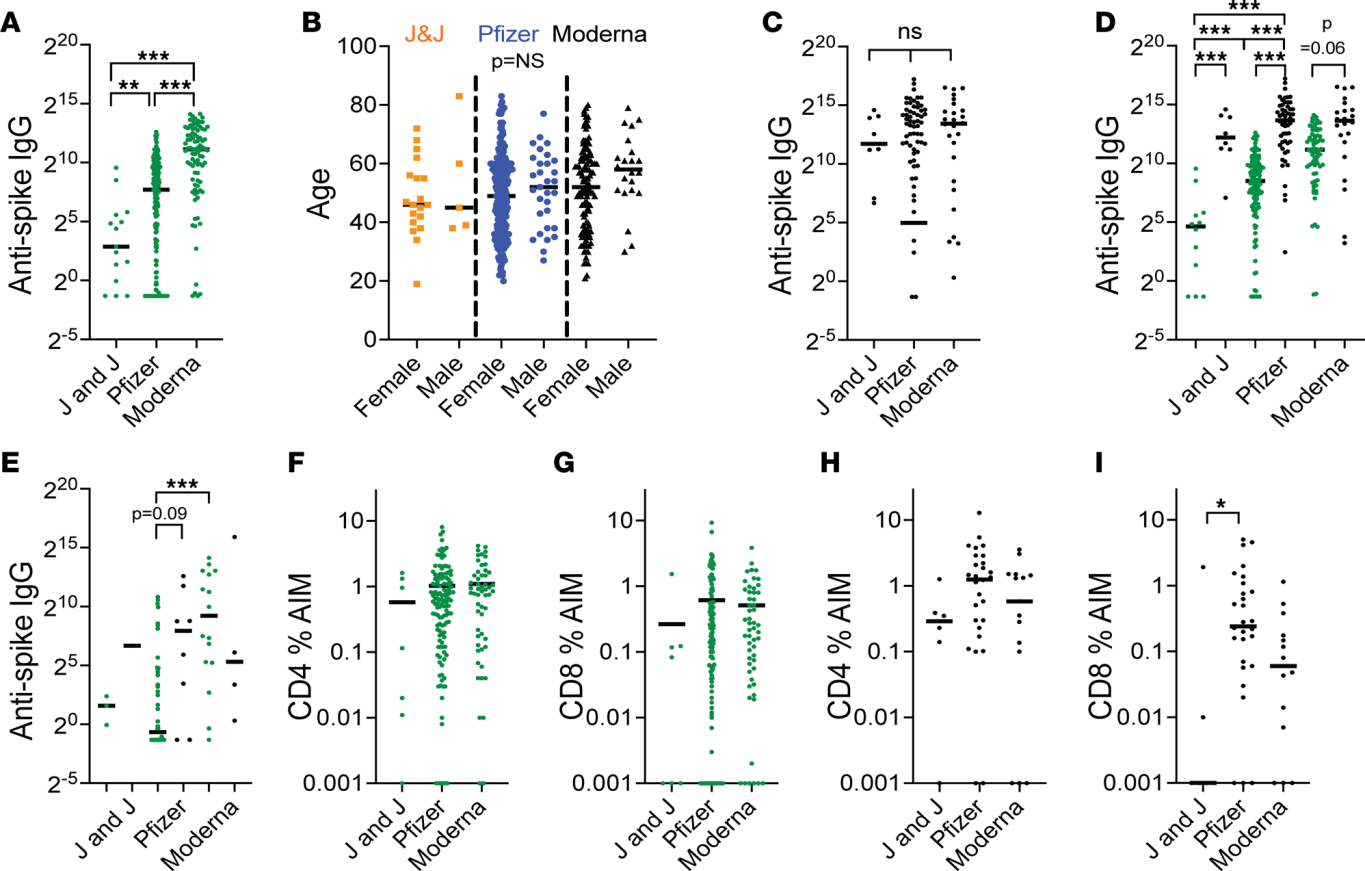
Adaptive immune responses following different SARS-CoV-2 vaccines in anti-NC^–^ (green symbols) and anti-NC^+^ (black symbols) patients. (**A** and **B**) Anti–spike IgG responses to different vaccines in anti-NC^–^ patients shows a better response to Moderna than to either Pfizer or Johnson & Johnson vaccines (**A**) that is not associated with differences in age or sex (**B**). (**C**) No difference in responses to the different vaccines in anti-NC^+^ patients. (**D** and **E**) Differences in anti-NC^–^ patients occur regardless of whether they were unexposed (**D**) or exposed (**E**) to B cell–depleting agents. (**F**–**I**) CD4 (**F** and **H**) and CD8 (**G** and **I**) T cell responses to SARS-CoV-2 spike peptides in anti-NC^–^ (**F** and **G**) and anti-NC^+^ (**H** and **I**) patients by vaccine type. Each data point represents an individual patient. Kruskal-Wallis ANOVA with Dunn’s correction for multiple comparisons. *P < 0.05, **P < 0.01, ***P < 0.001.

Of 234 patients with autoimmune diseases who received the BNT162b2 (Pfizer), 107 (45%) were NR at Post V1, compared with 15 of 24 patients (62%) who received Ad26.COV2.S (Johnson & Johnson) and 27 of 116 patients (23%) who received mRNA-1273 (Moderna). In anti-NC^–^ patients, those who received the Moderna vaccine had significantly higher anti–spike IgG values, compared with patients who received either the Pfizer (*P* < 0.001) or Johnson & Johnson (*P* < 0.001) vaccines (Figure 3A). This was not due to a difference in age or sex of the patients receiving the different vaccines (Figure 3B). There was no difference in anti–spike IgG values in anti-NC^+^ patients according to vaccine type received (Figure 3C). Of the 64 patients taking B cell depletion who received the Pfizer vaccine, 48 (75%) were NR, whereas 59 of 170 (34%) not receiving B cell depletion were NR. Anti-NC^–^ patients who received the Moderna vaccine had higher anti–spike IgG values at the Post V1 visit compared with those receiving the Pfizer vaccine, regardless of whether they were unexposed (Figure 3D) or exposed (Figure 3E) to B cell depletion. There were insufficient anti-NC^+^ patients taking B cell depletion to evaluate the differences between vaccine types (Figure 3E).

We evaluated the effect of stopping methotrexate and MMF on anti–spike IgG values. Methotrexate was held or stopped at initial vaccination in 11 patients, of whom 8 (73%) were Responders and 3 (27%) were NR. In 41 patients who continued methotrexate at Post V1, there were 28 of 41 (68%) Responders and 13 of 41 (32%) NR (*P* = 0.08). No differences were observed between patients who held or continued MMF or MPA during the initial vaccination series or MMF, MPA, or methotrexate at the time of boosting (Supplemental Table 4).

### T cell assays.

T cell assays were performed on matched samples from patients with autoimmune diseases at the Pre V, Post V1, and Post B1 visits and on HC at the Pre V and Post V1 visits (Figure 1). Samples with ≤ 1,000 CD4 T cells and/or ≤ 750 CD8 T cells were considered to have insufficient cells for accurate evaluation and were removed prior to analysis (Supplemental Figure 2, A and B). Vaccination of SARS-CoV-2–unexposed HC elicited robust T cell responses to SARS-CoV-2 peptides as expected (Supplemental Figure 2, C and D), with no effect on responses to CMV peptides (Supplemental Figure 2, E and F). Two healthy donors were used as controls for each batch and showed similar activation-induced marker (AIM) results over multiple blood draws (Supplemental Figure 2, G and H).

**Figure 4:**
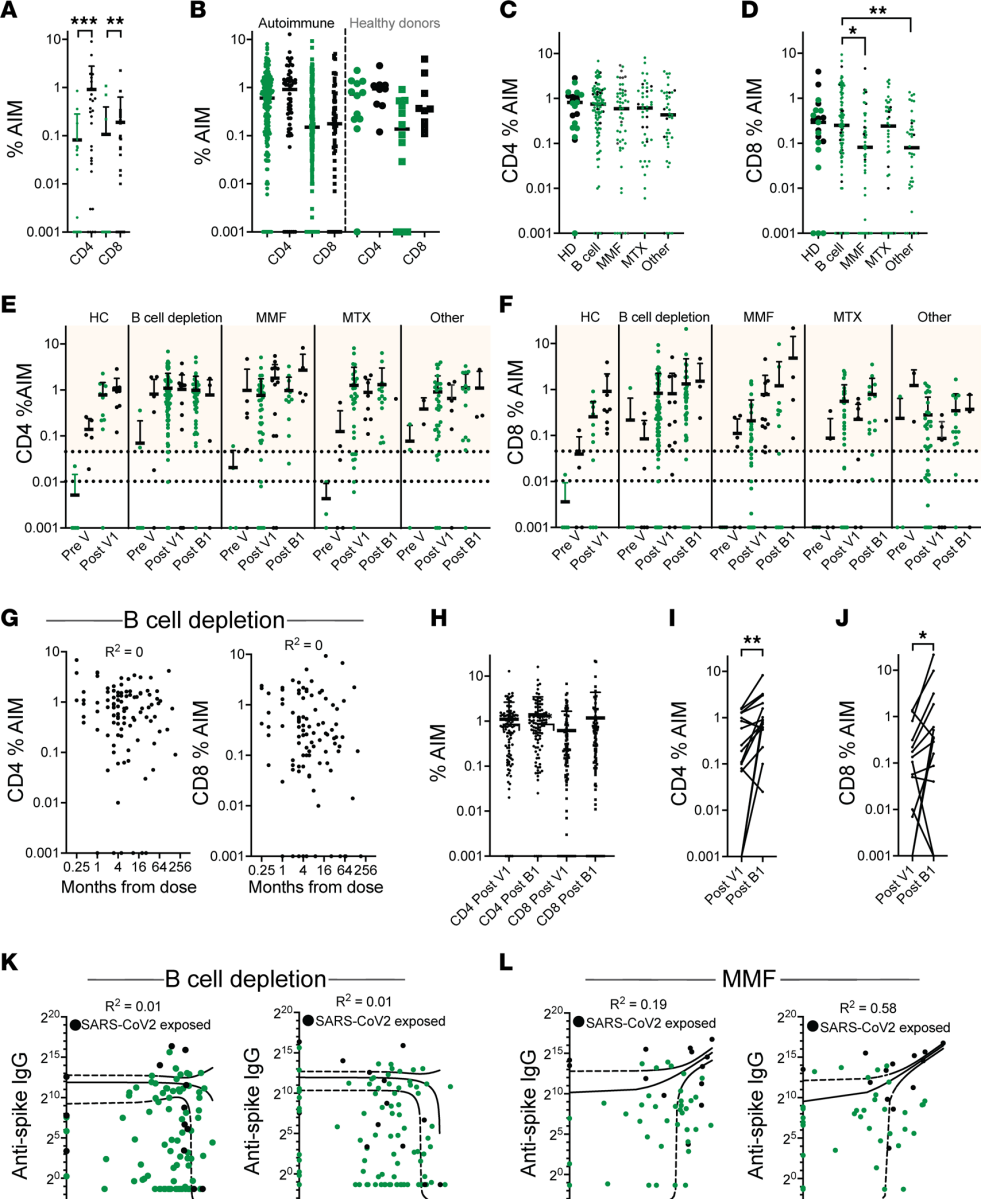
T cell responses to SARS-CoV-2 peptides. (**A**) CD4 and CD8 responses to spike peptides measured by activation-induced marker (AIM) assay in patients with autoimmune diseases before vaccination (**A**) and in HC and patients with autoimmune diseases at Post V1 (**B**) according to prior SARS-CoV-2 exposure. (**C** and **D**) CD4 (**C**) and CD8 (**D**) responses to spike peptides at Post V1 in patients with autoimmune diseases according to medication use. (**E** and **F**) CD4 (**E**) and CD8 (**F**) responses to spike peptides at sequential visits according to SARS-CoV-2 exposure and medication use. (**G**) No correlation between T cell responses to spike peptides at Post V1 and time since last dose of B cell depletion. (**H**) Change in CD4 and CD8 response to spike peptides after boosting in matched samples from the whole cohort. (**I** and **J**) Change in CD4 (**I**) and CD8 (**J**) response to spike peptides after boosting in matched samples from patients who were adherent to MMF treatment. (**K** and **L**) Correlation between T cell responses at Post V1 to spike peptides and anti–spike IgG values in patients treated with B cell depletion (**K**) or MMF (**L**). Each data point represents an individual patient. Anti-NC^–^ patients are shown as green symbols. Anti-NC^+^ patients are shown as black symbols. (**A** and **D**) Kruskal-Wallis ANOVA with Dunn’s correction for multiple comparisons. *P < 0.05, **P < 0.01, ***P < 0.001. (**I** and **J**) Mann Whitney *U* test. (**E**–**H**, **K**, and **L**) Univariable linear mixed regression.

We first analyzed T cell responses in all patients with autoimmune diseases using a linear regression model. At the Pre V visit, anti-NC^–^ patients had significantly lower CD4 and CD8 T cell reactivity to SARS-CoV-2 peptides than those patients with known SARS-CoV-2 exposure as expected (Figure 4A). Unlike what we observed with antibody responses, however, CD4 and CD8 responses to initial vaccination did not differ between patients with autoimmune diseases and HC or between SARS-CoV-2 exposed and nonexposed individuals (Figure 4B). The treatment analyses were, therefore, carried out regardless of exposure status. We analyzed T cell responses in patients taking B cell–depleting agents, MMF/MPA, methotrexate, or other drugs (Supplemental Table 5). The final linear regression model showed no difference in CD4 responses at the Post V1 visit among treatment groups (Figure 4, C and E; *P* = 0.86). By contrast, there was a significant difference in CD8 responses among treatment groups, (Figure 4, D and F; *P* = 0.013), reflecting a higher CD8 response in the B cell–depletion group compared with the MMF and other groups. No differences were observed in T cell responses to CMV peptides (Supplemental Figure 5, I and J). Unlike what we observed for anti–spike IgG, there was no relationship between the time of the last dose of B cell depletion and either the CD4 or CD8 response to SARS-CoV-2 peptides (Figure 4G).

The linear mixed regression model further showed no difference in the overall CD4 response between the Post V1 and Post B1 visits (Figure 4, E and H) but did show a statistically significant increase in the overall CD8 response after adjusting for treatment group (Figure 4, F and H; *P* = 0.002). When we examined the effect of the 4 different treatment regimens on booster responses, we found a significant increase in the CD4 response between the Post V1 and Post B1 visits only in the MMF group (*P* = 0.002), with a trend for CD8 (*P* = 0.01) but no significant differences in any of the other treatment groups (Figure 4, H–J).

We next determined whether there was a relationship between the anti–spike IgG antibody response and the T cell response to spike peptides at the Post V1 visit. We found no relationship between CD4 or CD8 responses and anti–spike IgG values in the B cell–depletion group (Figure 4K). However, in the final linear regression model, we found an inverse association between both CD4 and CD8 and anti–spike IgG among the small number of patients with B cell depletion who had previously been exposed to SARS-CoV-2 (*P* = 0.013 and *P* = 0.035, respectively). A similar inverse effect was observed for CD4 responses among SARS-CoV-2–exposed healthy donors (*P* = 0.017). By contrast, CD8 responses positively correlated with anti–spike IgG in patients on MMF regardless of SARS-CoV-2 exposure (*R*^2^ = 0.58, *P* = 0.014; Figure 4L).

Finally, we found no differences in the CD4 T cell response according to the vaccine received and only minimal differences in the anti-CD8 response (Figure 3, F–I).

### Disease diagnosis and vaccine response.

**Figure 5:**
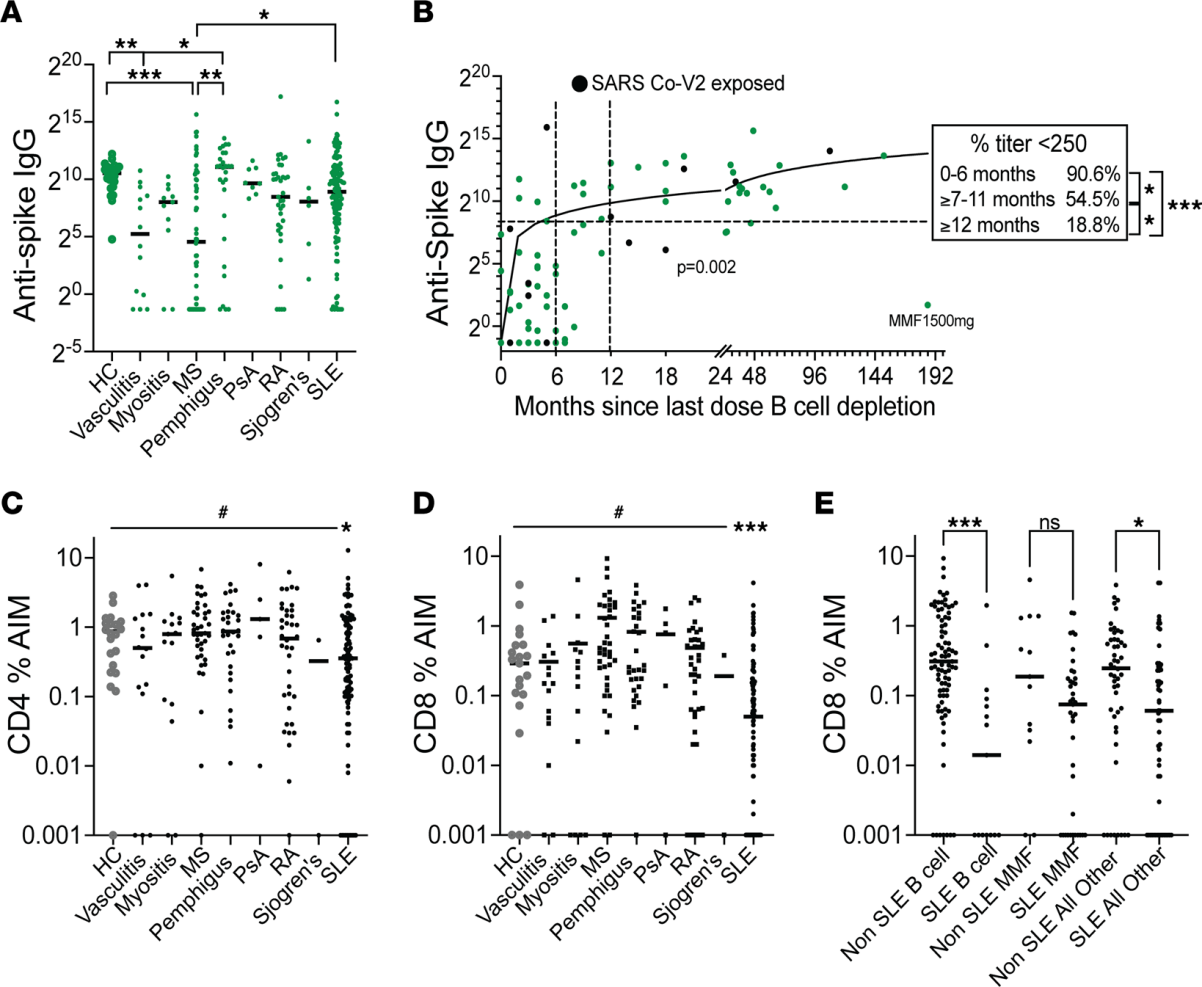
B and T cell responses to vaccination according to drug and diagnosis. (**A**) Anti–spike IgG values at Post V1 in anti-NC^–^ patients according to diagnosis. (**B**) Correlation of IgG anti-spike responses (U/mL) with time since last dose of B cell–depleting drug. Each data point represents an individual patient. Anti-NC^–^ patients are in green, and anti-NC^+^ patients are in black. Simple linear regression, *P* = 0.002. Inset shows percentage of nonresponders for each time window. (**C** and **D**) CD4 (**C**) and CD8 (**D**) T cell responses at the Post V1 visit according to diagnosis. Statistics for the linear mixed regression model (#) are shown in the bar. (**E**) Comparison of CD8 percentage of AIM responses in patients with and without SLE separated by those taking or not taking B cell–depleting drugs or MMF. Each data point represents an individual patient. Results of the linear mixed regression model are shown. *P < 0.05, **P <0.01, ***P < 0.001.

Anti-NC^–^ patients with multiple sclerosis (MS) had lower anti–spike IgG values at the Post V1 visit than anti-NC^–^ HC or patients with pemphigus or SLE (Figure 5A), most likely due to the high frequency of B cell depletion use in MS. In patients taking B cell–depleting drugs, the most important determinant of the anti–spike IgG response was the time since the last dose of the B cell–depleting drug. There was a significantly lower percentage of responders among those who received B cell depletion within the previous 6 months (9%), compared with those who received it ≥ 7–11 months (45%) or ≥ 12 months (81%) prior to vaccination (*P* < 0.05 and *P* < 0.001, respectively; Figure 5B). Patients with MS had taken their last dose of B cell depletion more recently than patients with pemphigus (median 5.5 versus 24 months since last dose, *P* < 0.0001). There were no differences in CD4 and CD8 responses at the Post V1 visit between patients with the most represented autoimmune diseases in our cohort, except for patients with SLE who had lower CD4 and CD8 responses than patients without SLE after correcting for medication use (*P* < 0.05 and *P* < 0.001, respectively; Figure 5, C and D). The difference in the CD8 response was still significant when patients with SLE and without SLE taking B cell–depleting drugs (*n* = 15 versus 85) or any other therapy except MMF/MPA (*n* = 56 versus 50) were compared (*P* < 0.001 and *P* < 0.05, respectively; Figure 5E), but the difference was not significant when patients with SLE and without SLE taking MMF (*n* = 40 versus 13) were compared (Figure 5E).

### Disease flares and vaccine type.

There were 16 disease flares (Supplemental Table 6) in 251 patients in whom flares were assessed at the Post V1 visit for an overall flare rate of 6.4%. The Post V1 flare rate in patients with SLE was 4 of 204 (2%), lower than reported in the literature (24, 25), and the flare rate in rheumatoid arthritis (RA) patients was 5 of 56 (9%) — similar to that reported in the literature (26, 27). There were 19 flares in 208 patients in whom flares were assessed after booster vaccination (Supplemental Table 6), for an overall flare rate of 9.1%. The Post B1 flare rate in patients with SLE was 8 of 71 (11%), similar to that reported in the literature, and the flare rate of RA patients was 5 of 22 (23%). The vaccine type was not associated with the rate of flares at either the Post V1 or Post B1 visit (Supplemental Table 7).

### Breakthrough infections.

SARS-CoV-2 infection frequency and severity at each visit are shown in Supplemental Table 8 and Supplemental Figure 3. At the time of the Pre V visit, 29.4% of the cohort had already been infected with SARS-CoV-2, with 2.1% requiring hospitalization. At the first follow-up visit (*n* = 253), 4.4% of patients had acquired a new infection, of whom only 1 was symptomatic. Patients seen for the first time 4–14 weeks after completion of the first vaccination series also had a high rate of previous exposure (14.6%); all but 2 of these infections were acquired before vaccination based on the timing of the sample collection in relation to completion of vaccination. The frequency of breakthrough infections was 4.8% at Post V2 but had increased to 25% by Post V3. By contrast, only 7.5% of patients receiving booster injections had breakthrough infections at the time of the Post B1 visit; the 1 patient in this group with a severe infection was hospitalized in the 2-week window immediately after receiving the booster. Medical charts of all patients who enrolled at the Pre V or Post V1 visit were examined regardless of whether they completed all visits, and there were no deaths due to SARS-CoV-2 in our cohort after 12 months of follow up, a period encompassing the Delta and Omicron variant waves.

The medications used by patients with breakthrough infections are listed in Supplemental Table 8. Sixty-five percent of breakthrough infections were symptomatic. The 2 hospitalized patients were both taking rituximab and had a negative anti–spike IgG titer. Patients with symptomatic infections were more often taking B cell–depleting agents (27% versus 17%), MMF (27% versus 17%), or azathioprine (15% versus 6%) compared with patients with asymptomatic infections, whereas those with asymptomatic infections were more often taking methotrexate (28% versus 6%) or hydroxychloroquine alone (17% versus 9%). There was insufficient power to determine the statistical significance of these findings. Thirty percent of patients with breakthrough infections had anti–spike IgG values of < 250 U/mL at the visit prior to the breakthrough infection, which was no different than the overall cohort. Sixty percent of patients with symptomatic infections prior to boosting had a preinfection anti–spike IgG titer of < 250 U/mL compared with 20% of patients with asymptomatic infections (*P* = 0.07). Similarly, 50% of patients with symptomatic infections after boosting had a preinfection anti–spike IgG value of < 250 U/mL compared with 22% of patients with asymptomatic infections. Notably, 9 patients failed to mount an anti-NC response to their breakthrough infections. Eight of 9 of these patients had anti–spike IgG values of < 250 U/mL, indicating that we may have underestimated the frequency of asymptomatic breakthrough infections in patients who did not mount IgG responses to vaccination.

Using a cutoff of 0.2 for a low CD4 response and 0.05 for a low CD8 response at the Post V1 visit, we found that 9 of 12 (75%) patients with autoimmune diseases with a breakthrough infection prior to boosting manifested low T cell responses at the Post V1 visit, compared with 90 of 228 (39%) of patients without breakthrough infections (*P* < 0.02). The other 3 patients, 1 of whom requiring hospitalization, had low anti–spike IgG values. Only 2 of 8 tested patients with breakthrough infections after boosting had low T cell responses; both had negative anti–spike IgG and symptomatic infections, and 1 required hospitalization.

### Autoantibodies.

**Figure 6:**
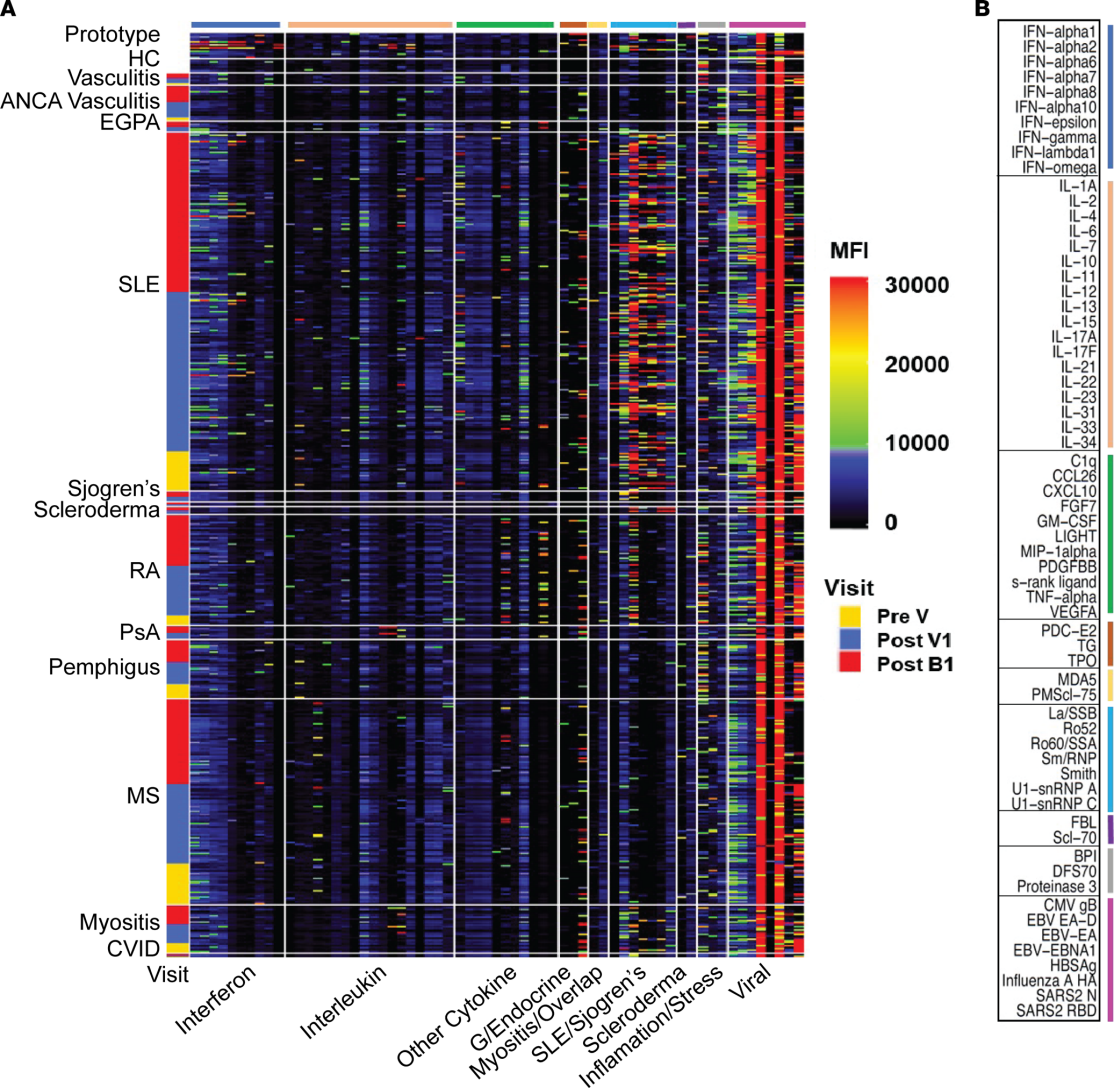
Autoantibody MFI values remain stable throughout vaccine course, with rare exceptions. (**A**) Heatmap representing serum IgG detected with an 83-plex array of cytokines and chemokines, traditional autoimmune-associated antigens, and viral antigens. Two hundred and forty-one vaccinated patients are represented and grouped into 1 of 15 different primary diagnoses. Within each diagnosis group, samples are clustered and annotated by the visit at which the sample was taken (Pre V, yellow; Post V1, blue; Post B1, red). Representative data from 16 prototype samples and 8 HC are included. ACE2 and CENPA were excluded from the analyses because of cross-reactivity. Only those analytes with values above 5,000 MFI are shown in the heatmap. Analytes in each group of antigens are color coded, and individual antigens in each group of antigens are shown in **B**. Comparisons were performed by either Mann-Whitney *U* test or linear mixed regression model.

To determine whether vaccination with spike protein induced new or increased titers of autoantibodies in individuals with autoimmune disease, autoantibody profiles were examined using a previously described autoantigen array (28) (Figure 6). As expected, patients with SLE displayed autoantibodies to nuclear antigens including SSA (Ro), SSB (La), Smith, and RNP, whereas patients with pemphigus and MS had few such autoantibodies. Antibodies against thyroid peroxidase (TPO) were common and were found across autoimmune diseases. Antibodies against type I IFNs, particularly IFN-α7 and -α8, were found most frequently in patients with SLE, whereas antibodies against IL-6 were most commonly found in patients with MS. Antibodies against TNF were detected in patients with RA who were taking TNF-inhibiting drugs, reflecting the circulating drugs in their serum.

We next examined autoantibody induction using matched patient sera before and after vaccination and boosting. We found no statistical difference in autoantibody median fluorescence intensity (MFI) for any analyte after vaccination or boosting, although a few patients had either an increase or decrease in the MFI of some autoantibodies.

Anti-SSA (Ro) antibodies have been reported in patients with severe SARS-CoV-2 infection (28, 29). We examined autoantibodies against Ro52 and Ro60 and La (Supplemental Figure 4, A–C). Autoantibodies against Ro60 and La remained stable over time. Although there was more variability in antibodies to Ro52, differences in MFI at the 3 time points were not significant. Autoantibodies against RNA-associated antigens Sm and RNP also remained stable (Supplemental Figure 4D). Two patients developed new autoantibodies to TPO after vaccination (Supplemental Figure 4E), confirmed by TPO ELISA, but neither developed thyroid dysfunction and 1 had a subsequent negative test after 24 months of follow up.

Autoantibodies to cardiolipin are induced by SARS-CoV-2 infection, and there was a significant incidence of thrombosis in patients infected during the initial waves of the pandemic (30–33). We therefore determined whether vaccination could induce or boost a preexisting anticardiolipin response. We found no difference in either IgM or IgG anti-CL titers between anti-NC^–^ and anti-NC^+^ patients at the prevaccination visit and no difference in titers between time points in the 2 groups (Supplemental Figure 4, F–I). Three patients had a modest increase in preexisting anti-CL antibodies, and 3 patients developed a de novo IgG or IgM anti-CL antibody of > 20 U. Of these, 1 with SLE had been previously positive, 1 with RA had a previous clot without anti-phospholipid antibodies, and 1 had eosinophilic granulomatosis with polyangiitis (EGPA). None of these patients developed thrombotic sequelae over the course of the study or at routine follow-up visits 24 months after initial vaccination.

Autoantibodies to desmogleins 1 and 3 were measured in paired serum samples from 23 pemphigus patients who received SARS-CoV-2 vaccination, including 14 patients with Pre V and Post V1 paired samples (10 PV, 4 PF) and 9 patients with Pre B and Post B1 paired samples (8 PV, 1 PF). Except for 1 patient who had a rising titer of anti-desmoglein antibodies prior to vaccination that continued after vaccination, there was no change in titers of autoantibodies to either antigen or development of a new autoantibody in this patient group after vaccination or boosting (Supplemental Figure 4, J and K).

Recent large studies using electronic medical data have suggested that there is an increase in the incidence of autoimmune diseases including RA, SLE, vasculitis, inflammatory bowel disease (IBD), and type 1 diabetes in SARS-CoV-2–exposed individuals (34). We therefore determined whether there was a difference in autoantibody profiles of patients with autoimmune diseases who had been previously exposed to SARS-CoV-2 compared with those who had not. We found no differences between these 2 groups (Supplemental Figure 5).

**Figure 7:**
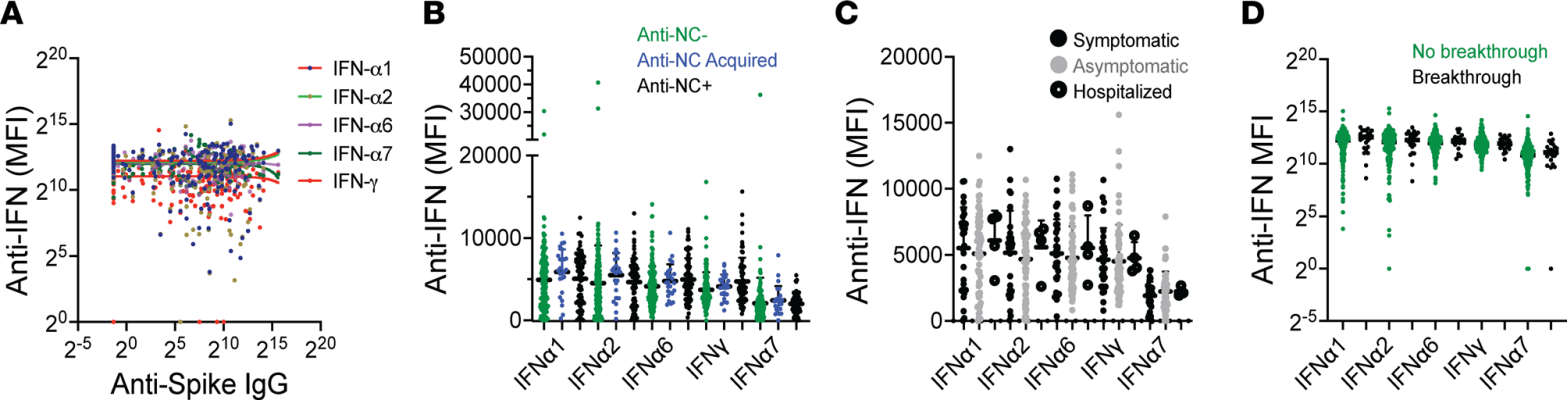
Correlation of autoantibodies to IFNs, measured at Post V1, with anti–spike IgG and SARS-CoV-2 infections. (**A**) No correlation of anti-IFN MFI with anti–spike IgG values. (**B**) No correlation of prior SARS-CoV-2 exposure with anti-IFN MFI. (**C**) No correlation of anti-IFN MFI with severity of prevaccination SARS-CoV-2 infections. (**D**) No correlation of anti-IFN MFI with frequency of breakthrough SARS-CoV-2 infections. Comparisons performed using Kruskal-Wallis ANOVA.

Antibodies to type I IFN have been associated with worse outcomes of SARS-CoV-2 infection (22). We therefore examined the correlation of antibodies to type I IFNs with the values of anti–spike IgG antibodies and with the severity of reported SARS-CoV-2 infections. There was no correlation between anti-IFN MFI and anti–spike IgG values (Figure 7A). Furthermore, there was no difference in anti-IFN MFI between anti-NC^–^ and anti-NC^+^ patients (Figure 7B) or between patients who had prevaccination symptomatic infections versus asymptomatic infections (Figure 7C). Furthermore, we found no difference in anti-IFN MFI between patients with or without breakthrough infections (Figure 7D).

### Unbiased predictive model for B and T cell responses to vaccination.

To identify additional variables associated with compromised B and T cell responses to vaccination, we developed predictive models of anti–spike IgG and T cell responses to the initial COVID-19 vaccination in an unbiased approach that included all the variables we recorded. The modeling was performed both with and without autoimmune diagnosis since the association of an autoimmune diagnosis with immune responses may be confounded by immunosuppressant use. The model that included autoimmune diagnosis had modest predictive power (*R*^2^ = 0.28) and identified the diagnosis of MS as a predictor of low anti–spike IgG response (Supplemental Figure 6A) except in patients treated with IFN-β or glatiramer acetate who mounted significantly higher anti–spike IgG values than the rest of the autoimmune cohort (*P* < 0.01), comparable with the HC (Supplemental Figure 6B). The model that excluded the autoimmune diagnosis also had modest predictive power (*R*^2^ = 0.25) and also found that IFN-β predicted a higher anti–spike IgG response at Post V1. In addition, B cell depletion or belimumab predicted a lower anti–spike IgG response (Supplemental Figure 6C). The models for CD4 and CD8 T cell response did not reveal additional meaningful associations beyond those already described.

## Discussion

Our real-world study yields a comprehensive overview of SARS-CoV-2 vaccine responses in a group of individuals representative of multiple autoimmune diseases compared with HC. We show significant differences in the B cell but not the T cell response to vaccination between SARS-CoV-2 naive and preexposed patients, discordance between anti–spike IgG antibody and T cell responses in patients with different autoimmune diseases, and minimal effects of vaccination on autoantibody and anticytokine reactivities after vaccination.

Using the Roche Elecsys assay to evaluate the anti–spike IgG protein response after vaccination, we established that thresholds for a normal response depend on prior SARS-CoV-2 exposure. Response rates below the cutoff values after the first vaccination occurred in 25% and 44% of exposed and unexposed cohorts, respectively. Dissipation of the humoral immune response over time and response to boosting were similar in patients with autoimmune diseases and controls.

Medication use was a major determinant of low antibody response in patients with autoimmune diseases. Consistent with prior literature, most patients with an inadequate humoral response to both the initial and booster vaccinations were taking B cell–depleting drugs (5, 7, 8, 35, 36), with the time from the last dose of B cell depletion being a critical factor (37). There were low humoral responses in > 50% of patients even in the 6- to 12-month window after the last dose of the B cell–depleting drug. These findings have general implications for the immunization of patients taking B cell–depleting drugs for whom current guidelines recommend a window of 6 months after treatment or 4 weeks prior to the next treatment cycle for delivery of vaccines (38). Other immunosuppressive agents that conferred a high frequency of suppression of anti–spike IgG responses were the combination of MMF and belimumab and treatment with fingolimod. Because we assessed MMF levels and found that approximately 20% of patients were not taking their medication, we were able to evaluate a true rate of low IgG response in patients taking MMF without an additional immunosuppressant at 39%, higher than previously reported (4, 7, 8). Most patients did not contact their physicians or hold their medications prior to vaccination. Methotrexate was held most frequently with a trend for an improved vaccine response, consistent with recent clinical trials (12, 13, 39). We were unable to show that holding MMF improved vaccine responses. Few of our patients were taking high doses of steroids, but doses of < 20 mg/day were not associated with low IgG responses. These findings identify those patients at highest risk of nonresponse to vaccination.

The vaccine type also influenced immune responses in our patients. In a previous study of patients with autoimmune diseases in the United Kingdom, the viral vector vaccine ChAdOx1 nCoV-19 was less effective than the Pfizer vaccine at eliciting anti–spike IgG responses, but it induced a stronger T cell response (23). In our study, differences between vaccines were observed only in SARS-CoV-2–naive patients. Patients immunized with the Johnson & Johnson vaccine mounted lower IgG responses than patients immunized with mRNA vaccines. Notably, the anti–spike IgG response was higher in patients immunized with the Moderna vaccine than in those immunized with the Pfizer vaccine even after adjusting for usage of B cell–depleting drugs. A recent metaanalysis of observational studies of immunocompromising conditions, including autoimmune disease, found that the Moderna vaccine was associated with reduced risk of SARS-CoV-2 infection hospitalization and mortality compared with the Pfizer vaccine (40). Additionally, the Moderna vaccine was associated with increased seroconversion rates compared with the Pfizer vaccine in immunosuppressed transplant recipients (41). Increased TLR stimulation with the higher-dose Moderna vaccine, compared with the Pfizer vaccine, has been hypothesized to lead to improved seroconversion in immunocompromised patients, which may account for the differences in conversion rates found in this study and others (42, 43). Alternatively, differences in formulation or in the prime-boost timing of the 2 vaccines could contribute to differences in efficacy. The vaccine type had much less effect on the quality of the T cell response, and we could not confirm a previously reported higher T cell response to the Moderna compared with the Pfizer vaccine (44).

We observed differential effects of immunosuppression on B and T cell responses across various classes of immunosuppressive drugs. Consistent with prior findings, patients with MS taking B cell–depleting drugs exhibited lower anti–spike IgG responses and higher CD8 responses to spike protein peptides (14, 16, 17, 36, 45). This heightened CD8 response could result from reduced anti–spike IgG levels in B cell–depleted patients, prolonging spike protein clearance after vaccination. Conversely, MMF usage coordinately suppressed both B and T cell responses, reflecting the drug’s antiproliferative activity.

While differences in the humoral response to vaccine between patients with different autoimmune diseases could be attributed to medications, we found that the CD8 response to vaccine was lower in patients with SLE than in HC and in patients with other autoimmune diseases, including those receiving B cell–depleting drugs. Comparable results comparing patients with SLE with HC have been recently reported (46). Mitochondrial and metabolic defects in T cell function occur in SLE; these defects are more pronounced in CD8 than in CD4 T cells and are associated with an increased risk of recurrent infections (47, 48). Furthermore, a subset of patients with SLE has a profile of CD8 T cell exhaustion, a phenotype that has been associated with worse response to vaccinations and viral infections (49).

Despite the relatively high occurrence of subnormal vaccination responses, severe breakthrough infections were rare in our cohort. Importantly, unlike a recent report from a similar United Kingdom cohort (23), we had no deaths among our immunosuppressed patients. This discrepancy likely arises from the United Kingdom cohort’s inclusion of more vulnerable individuals. Moreover, the overall frequency of breakthrough infections in patients with autoimmune diseases was lower than in HC and was not higher in patients with SLE than in patients with other autoimmune diseases despite their CD8 T cell defect. The reasons for this difference are multifactorial and could include the earlier immunization time frame of our autoimmune cohort relative to the pandemic’s evolution as well as variations in the prevalent variant during immunization and boosting. Additionally, cautious behavior among patients with autoimmune diseases might have contributed as may as the utilization of tixagevimab with cilgavimab (Evusheld), although this drug received emergency use authorization in December 2021 and was prescribed to only 3 patients in our cohort during the study period. Protection of our patients was sustained until the second postvaccination visit but waned by the third (12-month) visit in patients who did not receive a timely dose of the booster.

Data on the association between anti–type I IFN antibodies and SARS-CoV-2 infection to date are mixed (22, 50), likely as a consequence of differing characteristics of individual cohorts. Our data show that anti-cytokine antibodies do not correlate either with the magnitude of the immune response to vaccination or the frequency or severity of breakthrough infections in patients with autoimmune diseases.

Pre–SARS-CoV-2–era studies in patients with autoimmune diseases have demonstrated minimal induction of new autoantibodies by vaccines or adjuvants (51). Large-cohort studies have linked SARS-CoV-2 infection to autoantibody induction and increased risk of new-onset autoimmune disease (34, 52, 53). The SARS-CoV-2 mRNA vaccines contain pseudouridine nucleoside–modified mRNA designed for reduced inflammation compared with unmodified RNA. Nevertheless, these vaccines still activate the MDA5/type I IFN innate immune pathway, leading to CD8 T cell response and production of inflammatory chemokines and cytokines like IL-1 and IFN-γ along with potent activation of Tfh cell and germinal center responses (54, 55). Furthermore, observational studies have reported a variable rate of disease flares ranging from 0.4% to 20% following vaccination (56). We were therefore particularly interested in whether vaccination in the context of an innate immune stimulus would induce an increase in existing autoantibody MFI or new autoantibodies or disease flares. Reassuringly, although a few patients developed new autoantibodies to TPO and cardiolipin without clinical autoimmune disease, we did not observe emergence of new specificities or significant increases in autoantibodies in our array panel or in either anti-cardiolipin or anti-desmoglein antibodies by ELISA. In accordance with these data, disease flares of either RA or SLE did not occur at a higher frequency than reported in the literature, and there was no association of vaccine type with disease flares.

This real-world study has several strengths. The study encompassed multiple autoimmune diseases spanning rheumatologic, neurologic, and dermatologic domains, and it included patients of diverse race, sex, and age. By using matched pre- and postvaccine samples, we were able to analyze the effect of SARS-CoV-2 vaccination on existing autoimmunity, including the potential elicitation of new autoantibodies and the effects of anti-cytokine antibodies on vaccine responses. The study involved patients on various immune-suppressive medications, including conventional immunosuppressives and biologics, confirming previously described medication effects on vaccine responses while evaluating both B and T cell responses concurrently. In a reflection of the real-world scenario, the study included patients who interrupted and those who did not interrupt their immunosuppressive medications.

There were also some limitations to the study, notably the challenge of small sample sizes for some of the subanalyses. Additionally, the interruption of medications lacked standardization, reflecting how patients obtained their vaccines (often without first contacting their physicians). The nonrandomized interruption of medications introduced potential confounding factors, such as disease activity and patient beliefs. The evolution of SARS-CoV-2 variants and the compromise of antiviral responses by some immunosuppressive regimens may also have affected our analysis of the frequency of asymptomatic breakthrough infections. Moreover, dropouts and assessments occurring outside of the timing window added complexities to the study’s interpretation. Finally, we used the anti–spike IgG and anti-IFN values as measurements of the antibody response without concomitant neutralization assays. This may have resulted in overestimation of the quality of the response in patients with autoimmune diseases in which the neutralization capacity of anti–spike IgG may be compromised (46) and there may be an underestimation of the effect of cytokine neutralization on anti–spike IgG responses.

Overall, our study provides data on factors associated with poor B and T cell responses to SARS-CoV-2 vaccination in patients with autoimmune diseases. The significantly enhanced responsiveness to vaccination among patients previously exposed to SARS-CoV-2 virus infection indicates that most patients with autoimmune diseases can mount antiviral responses. This suggests that safe immunization strategies for autoimmune populations could include increased mRNA vaccine dose, extra boosting, or boosting with a wider range of viral antigens. Using both clinical and laboratory-based measures, we offer reassuring data regarding the minimal induction of autoimmunity by SARS-CoV-2 vaccines. We also demonstrate little effect of anti-cytokine responses on vaccine response or breakthrough infections. Importantly, we show that B and T cell responses to vaccination are not always correlated and that T cell responses, except in patients with SLE, resemble those in HC with enhanced CD8 but not CD4 responses after boosting. Despite the high rate of compromised anti–spike IgG responses, the low rate of breakthrough infections in our cohort provides additional support for the protective function of vaccine-induced T cell responses across a broad swath of patients with autoimmune diseases.

## Methods

### Patient enrollment and sex as a biological variable

Clinical data and biospecimens were obtained from a prospective observational study involving 75 male and 388 female patients aged ≥ 18 years with autoimmune diseases at 4 NIH Autoimmunity Centers of Excellence (Feinstein Institutes for Medical Research, University of Pennsylvania, Oklahoma Medical Research Foundation, and Emory University) from January 2021 to September 2022. Males and females behaved similarly (Figure 3B and Supplemental Figure 1D) and are reported together. The autoimmune diseases studied are shown in Figure 1. Clinical assessments (see Supplemental Methods) occurred before receiving an mRNA or vector-based SARS-CoV-2 vaccination (Pre V) and at subsequent postvaccine visits. Post V1, V2, and V3 occurred at 4–14 weeks, 24 ± 8 weeks, and 52 ± 8 weeks, respectively, after completion of full vaccination (1 dose for protein vaccine or 2 doses for mRNA vaccines). Additional assessments occurred before the first booster for newly enrolled patients (Pre B) and 2–8 weeks after receiving the first SARS-CoV-2 booster (Post B1). One hundred and twelve sera from healthy donors, matched by age, sex, and ethnicity to the autoimmune cohort, were obtained from the Serological Sciences Network for COVID-19 (Seronet) database (57) (Figure 1) during the same time windows relative to vaccination. Samples were collected from 2 separate cohorts of HC: one with matched Pre V and Post V1 visits and the other with matched Pre B and Post B1 visits. Control peripheral blood mononuclear cells (PBMCs) were processed from whole blood collected from healthy donors at the Feinstein Institutes for Medical Research and Emory University, and pre–COVID-era serum samples were obtained from Stanford University.

Chart reviews to evaluate SARS-CoV-2–related deaths were conducted on all patients a minimum of 1 year after initial vaccination regardless of inclusion in the analyses.

### Serologic testing

IgG antibodies to SARS-CoV-2 spike and NC proteins were measured at Northwell Core Laboratories (New Hyde Park, New York, USA) using the Roche Elecsys assay (Roche Diagnostic Corporation), with serial 10-fold dilutions in Roche assay buffer if the initial anti–spike IgG value was > 250 U/mL. Anti-NC antibodies were considered positive when the assay result was > 0.5 U/mL for patients with autoimmune diseases and > 0.8 U/mL for healthy donors, as these sera were diluted 1:2 for the assay with 0.4 U/mL being the lower limit. Antibody details are provided in Supplemental Tables 9 and 10.

### MMF or MPA levels

For those patients being prescribed MMF or MPA, sera from the Post V1 and/or Post B1 visits were tested for drug levels using a commercial assay (Labcorp). Patients with values of < 5 μg/mL were considered nonadherent.

### T cell activation-induced marker (AIM) assays and flow cytometry

CD4 and CD8 responses to SARS-CoV-2 peptides were assessed using AIM flow cytometric assays employing the following 3 activation markers: CD137, Ox40, and CD69. Cells were stimulated for 20–24 hours at 37°C in 1 of 4 conditions: (a) vehicle control, (b) anti-CD3/CD28 beads, (c) PepTivator CMV pp65, or (d) PepTivator SARS-CoV-2 Prot_S Complete (Supplemental Methods). The gating strategy for identifying B and T cells and the distribution of immune cell counts is shown in Supplemental Figure 7. After gating on CD4 or CD8, the percent AIM^+^ values were calculated using Boolean “OR” gating as follows: CD137^+^OX40^+^ “OR” CD137^+^CD69^+^ “OR” OX40^+^CD69^+^. The vehicle control percent AIM+ values were subtracted from each stimulation percent AIM+ value to control for nonspecific background activation. Representative FACS plots and AIM assay gating are shown in Supplemental Figure 8.

### Autoantibodies

An 83-plex custom, bead-based antigen array consisted of 3 broad categories of antigens (Supplemental Table 9). Each array was constructed as previously described (28) by conjugating antigens to uniquely barcoded, carboxylated magnetic beads (MagPlex-C, Luminex Corp.; Supplemental Methods). The “Cytokine” content included 49 cytokines, chemokines, growth factors, acute phase proteins, and cell surface proteins. The “Traditional Autoimmune Associated” content included 21 commercial protein antigens associated with connective tissue diseases. The “Viral” content included 8 antigens derived from viruses such as SARS-CoV-2, respiratory syncytial virus (RSV), and cytomegalovirus (CMV).

Samples were initially run in singlet, and selected samples were rerun in duplicate to confirm significant changes in autoantibody levels. Binding events were displayed as MFI. For each sample, MFI values for “bare bead” IDs were subtracted from the MFI values for each antigen-conjugated bead ID. To normalize across samples, the median MFI values for the 4 control IgG analytes (anti–human IgG [H+L], anti–human IgG F[ab’] fragment–specific, anti–human IgG Fc fragment–specific, and human IgG from serum) were calculated. For each of the control IgG analytes, the ratio of the MFI value for each sample to the corresponding median was then calculated. The average of these 4 ratios became the correction factor for all the analytes of that sample in that the MFI of each analyte was divided by the correction factor.

IgG and IgM antibodies to cardiolipin were measured by the College of American Pathologists/Clinical Laboratory Improvement Amendments (CAP/CLIA) certified Oklahoma Medical Research Foundation Clinical Immunology Laboratory (58). Antibodies to desmoglein 1 (pemphigus foliaceus) and desmoglein 3 (pemphigus vulgaris) were measured by commercial ELISA (Euroimmun, EA1495-4801 G and EA1496-4801 G) using serial dilutions of serum samples within the linear range of standard controls (59, 60). Corrected index values were calculated by multiplying index values by the dilution factor.

### Statistics

Descriptive statistics (frequency distribution for categorical variables and mean, SD, median, interquartile range, minimum, and maximum for continuous variables) were calculated.

#### Anti–spike IgG antibodies.

Wilcoxon signed-rank test was used to determine whether there was a change in anti–spike IgG between Post V1 and Post V2 visits or Pre B and Post B1 visits. The Mann-Whitney *U* test or Kruskal-Wallis ANOVA was used to determine whether there was a difference in the change in anti–spike IgG from Pre V to Post V1 visits, Post V1 to Post V2 visits, or Pre B to Post B1 visits among autoimmune or HC. Dwass, Steel, Critchlow-Fligner (DSCF) adjustment was performed to adjust for multiple comparisons. Kruskal-Wallis ANOVA was used to determine whether there was a difference in the change in anti–spike IgG between anti-NC^–^, anti–NC Acq, and anti-NC^+^ patients at each visit.

#### T cell assays.

Univariable linear mixed regression was used to screen variables (age, sex, race, ethnicity) with a *P* value criterion of *P* < 0.05 for entry into the model selection procedure. Backward selection was used with variable entry and retention criteria of *P* < 0.05 to select the final multivariable model. Linear mixed model was performed to determine whether there was a change in CD4 or CD8 response between Pre V and Post V1 visits, a difference in CD4 or CD8 response between anti-NC^–^ and anti-NC^+^ patients, a correlation between CD4/8 responses and anti–spike IgG responses at Post V1 among the treatment groups in all patients and in anti-NC^+^ or anti-NC^–^ patients, or a difference in CD4 or CD8 response among groups. Log transformation was applied to meet the required assumptions of the regression model. Interaction between treatment groups and SARS-CoV-2 exposure status was examined. Tukey’s exact procedure was performed to adjust for multiple comparisons. Additional regression analyses were performed using Prism 9.0.

#### Autoantibodies.

Differences between autoantibody MFI at the Pre V and Post V1 or Post B1 time points were analyzed by Mann-Whitney *U* test for 2 time points or linear mixed model for 3 time points.

#### Unbiased predictive models of anti–spike IgG antibody and T cell response.

Linear regression modeling was used to determine correlations of anti–spike IgG values, CD4 T cell percentage of AIM, or CD8 T cell percentage of AIM at the Post V1 visit with other serological and clinical variables. See Supplemental Methods for extended method.

### Study approval

This research was approved by each center’s IRBs of Feinstein Institutes of Medical Research, University of Pennsylvania, OMRF and Emory University; written informed consent was obtained from each patient prior to performance of any study procedures.

### Data availability

Data are available in the Supporting Data Values file and the Datasets file.

## Author contributions

Design of research studies was contributed by EA, MP, GM, SN, MM, BD, TPZ, A Khosroshahi, IS, ETLP, ABO, JJ, JMB, AP, PJU, CA, and AD. Recruitment of patients was contributed by GM, SN, PKG, MM, A Khosroshahi, ABO, JJ, AP, CA, and AD. Experiments were conducted by MP, EY, A Kar, RM, AC, CE, NCO, EJC, CA, GP, JG, JJ, AP, PJU, and JMB. Data were acquired by MP, EY, A Kar, NCO, EJC, JJ, PJU, JMB, and AD. Data were analyzed by EA, MP, EY, A Kar, TML, CS, RS, JZ, TL, TPZ, AP, PJU, JMB, and AD. Reagents were provided by PKG, JJ, AP, PJU, and JMB. Scripts for predictive model building were provided by RS, TL, CS, TL, and TPZ. The manuscript was written by EA, MP, EY, RS, TML,TL, CA, and AD. The manuscript was edited by BD, ETLP, ABO, JJ, JM, PJU, and JMB.

## Supplementary Material

Supplemental data

Supplemental data set 1

Supporting data values

## Figures and Tables

**Table 1:**
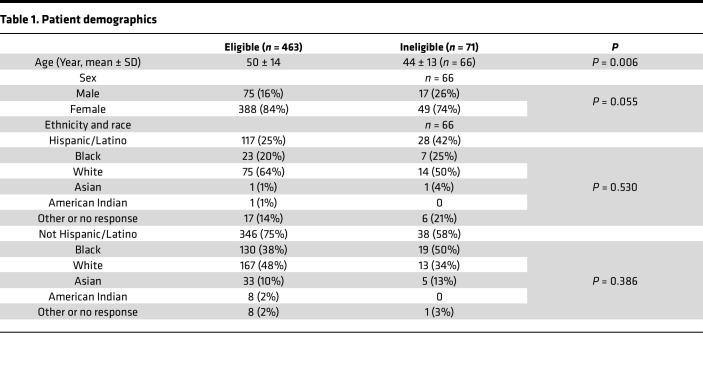
Patient demographics

## Version 1 06/04/2024

In-Press Preview

## Version 2 07/08/2024

Electronic publication

## References

[1] Akiyama S (2021). Prevalence and clinical outcomes of COVID-19 in patients with autoimmune diseases: a systematic review and meta-analysis. Ann Rheum Dis.

[2] Guimarães LE (2015). Vaccines, adjuvants and autoimmunity. Pharmacol Res.

[3] Teijaro JR, Farber DL (2021). COVID-19 vaccines: modes of immune activation and future challenges. Nat Rev Immunol.

[4] Zecca E (2022). Ongoing mycophenolate treatment impairs anti-SARS-CoV-2 vaccination response in patients affected by chronic inflammatory autoimmune diseases or liver transplantation recipients: results of the RIVALSA prospective cohort. Viruses.

[5] Spiera R (2021). Rituximab, but not other antirheumatic therapies, is associated with impaired serological response to SARS- CoV-2 vaccination in patients with rheumatic diseases. Ann Rheum Dis.

[6] Haberman RH (2021). Methotrexate hampers immunogenicity to BNT162b2 mRNA COVID-19 vaccine in immune-mediated inflammatory disease. Ann Rheum Dis.

[7] Furer V (2021). Immunogenicity and safety of the BNT162b2 mRNA COVID-19 vaccine in adult patients with autoimmune inflammatory rheumatic diseases and in the general population: a multicentre study. Ann Rheum Dis.

[8] Ferri C (2021). Impaired immunogenicity to COVID-19 vaccines in autoimmune systemic diseases. High prevalence of non-response in different patients’ subgroups. J Autoimmun.

[9] De Santis M (2022). Dose-dependent impairment of the immune response to the Moderna-1273 mRNA vaccine by mycophenolate mofetil in patients with rheumatic and autoimmune liver diseases. Vaccines (Basel).

[10] Bugatti S (2021). Methotrexate and glucocorticoids, but not anticytokine therapy, impair the immunogenicity of a single dose of the BNT162b2 mRNA COVID-19 vaccine in patients with chronic inflammatory arthritis. Ann Rheum Dis.

[11] Frommert LM (2022). Type of vaccine and immunosuppressive therapy but not diagnosis critically influence antibody response after COVID-19 vaccination in patients with rheumatic disease. RMD Open.

[12] Abhishek A (2022). Effect of a 2-week interruption in methotrexate treatment versus continued treatment on COVID-19 booster vaccine immunity in adults with inflammatory conditions (VROOM study): a randomised, open label, superiority trial. Lancet Respir Med.

[13] Arumahandi de Silva AN (2022). Pausing methotrexate improves immunogenicity of COVID-19 vaccination in elderly patients with rheumatic diseases. Ann Rheum Dis.

[14] Mrak D (2021). SARS-CoV-2 vaccination in rituximab-treated patients: B cells promote humoral immune responses in the presence of T-cell-mediated immunity. Ann Rheum Dis.

[15] Mahil SK (2021). The effect of methotrexate and targeted immunosuppression on humoral and cellular immune responses to the COVID-19 vaccine BNT162b2: a cohort study. Lancet Rheumatol.

[16] Bonelli MM (2021). SARS-CoV-2 vaccination in rituximab-treated patients: evidence for impaired humoral but inducible cellular immune response. Ann Rheum Dis.

[17] Bitoun S (2022). Rituximab impairs B cell response but not T cell response to COVID-19 vaccine in autoimmune diseases. Arthritis Rheumatol.

[18] Wherry EJ, Barouch DH (2022). T cell immunity to COVID-19 vaccines. Science.

[19] Zonozi R (2023). T cell responses to SARS-CoV-2 infection and vaccination are elevated in B cell deficiency and reduce risk of severe COVID-19. Sci Transl Med.

[20] Wang X (2023). Autoantibodies against type I interferons in COVID-19 infection: a systematic review and meta-analysis. Int J Infect Dis.

[21] Chauvineau-Grenier A (2022). Autoantibodies neutralizing type I interferons in 20% of COVID-19 deaths in a French hospital. J Clin Immunol.

[22] Bastard P (2020). Autoantibodies against type I IFNs in patients with life-threatening COVID-19. Science.

[23] Barnes E (2023). SARS-CoV-2-specific immune responses and clinical outcomes after COVID-19 vaccination in patients with immune-suppressive disease. Nat Med.

[24] Furie R (2021). Anifrolumab reduces flare rates in patients with moderate to severe systemic lupus erythematosus. Lupus.

[25] McElhone K (2021). Flares in patients with systemic lupus erythematosus. Rheumatology (Oxford).

[26] Raheel S (2017). Improved flare and remission pattern in rheumatoid arthritis over recent decades: a population-based study. Rheumatology (Oxford).

[27] Markusse IM (2015). Disease flares in rheumatoid arthritis are associated with joint damage progression and disability: 10-year results from the BeSt study. Arthritis Res Ther.

[28] Chang SE (2021). New-onset IgG autoantibodies in hospitalized patients with COVID-19. Nat Commun.

[29] Fujii H (2020). High levels of anti-SSA/Ro antibodies in COVID-19 patients with severe respiratory failure: a case-based review: High levels of anti-SSA/Ro antibodies in COVID-19. Clin Rheumatol.

[30] Zhang Y (2020). Coagulopathy and Antiphospholipid Antibodies in Patients with Covid-19. N Engl J Med.

[31] Trahtemberg U (2021). Anticardiolipin and other antiphospholipid antibodies in critically ill COVID-19 positive and negative patients. Ann Rheum Dis.

[32] Bertin D (2022). Anti-cardiolipin IgG autoantibodies associate with circulating extracellular DNA in severe COVID-19. Sci Rep.

[33] Bertin D (2020). Anticardiolipin IgG autoantibody level is an independent risk factor for COVID-19 severity. Arthritis Rheumatol.

[34] Sharma C, Bayry J (2023). High risk of autoimmune diseases after COVID-19. Nat Rev Rheumatol.

[35] Boekel L (2021). Antibody development after COVID-19 vaccination in patients with autoimmune diseases in the Netherlands: a substudy of data from two prospective cohort studies. Lancet Rheumatol.

[36] Apostolidis SA (2021). Cellular and humoral immune responses following SARS-CoV-2 mRNA vaccination in patients with multiple sclerosis on anti-CD20 therapy. Nat Med.

[37] Moor MB (2021). Humoral and cellular responses to mRNA vaccines against SARS-CoV-2 in patients with a history of CD20 B-cell-depleting therapy (RituxiVac): an investigator-initiated, single-centre, open-label study. Lancet Rheumatol.

[38] Kant S (2021). Timing of COVID-19 vaccine in the setting of anti-CD20 therapy: a primer for nephrologists. Kidney Int Rep.

[39] Araujo CSR (2022). Two-week methotrexate discontinuation in patients with rheumatoid arthritis vaccinated with inactivated SARS-CoV-2 vaccine: a randomised clinical trial. Ann Rheum Dis.

[40] Wang X (2023). Comparative effectiveness of mRNA-1273 and BNT162b2 COVID-19 vaccines in immunocompromised individuals: a systematic review and meta-analysis using the GRADE framework. Front Immunol.

[41] Boyarsky BJ (2021). Antibody response to a single dose of SARS-CoV-2 mRNA vaccine in patients with rheumatic and musculoskeletal diseases. Ann Rheum Dis.

[42] Mues KE (2022). Real-world comparative effectiveness of mRNA-1273 and BNT162b2 vaccines among immunocompromised adults identified in administrative claims data in the United States. Vaccine.

[43] Dickerman BA (2022). Comparative effectiveness of BNT162b2 and mRNA-1273 vaccines in U.S. veterans. N Engl J Med.

[44] Chung A (2022). Antibody and T-cell responses by ultra-deep T-cell receptor immunosequencing after COVID-19 vaccination in patients with plasma cell dyscrasias. Br J Haematol.

[45] Prendecki M (2021). Humoral and T-cell responses to SARS-CoV-2 vaccination in patients receiving immunosuppression. Ann Rheum Dis.

[46] https://www.ncbi.nlm.nih.gov/pmc/articles/PMC10312827/.

[47] Buang N (2021). Type I interferons affect the metabolic fitness of CD8^+^ T cells from patients with systemic lupus erythematosus. Nat Commun.

[48] Chen PM (2022). CD38 reduces mitochondrial fitness and cytotoxic T cell response against viral infection in lupus patients by suppressing mitophagy. Sci Adv.

[49] McKinney EF (2015). T-cell exhaustion, co-stimulation and clinical outcome in autoimmunity and infection. Nature.

[50] Wolff ASB (2023). Vaccination prevents severe COVID-19 outcome in patients with neutralizing type 1 interferon autoantibodies. iScience.

[51] Guimarães LE (2015). Vaccines, adjuvants and autoimmunity. Pharmacol Res.

[52] Tesch F (2023). Incident autoimmune diseases in association with SARS-CoV-2 infection: a matched cohort study. Clin Rheumatol.

[53] Lim SH (2023). Autoimmune and autoinflammatory connective tissue disorders following COVID-19. JAMA Netw Open.

[54] Verbeke R (2022). Innate immune mechanisms of mRNA vaccines. Immunity.

[55] Li C (2022). Mechanisms of innate and adaptive immunity to the Pfizer-BioNTech BNT162b2 vaccine. Nat Immunol.

[56] Xie Y (2022). The flare of rheumatic disease after SARS-CoV-2 vaccination: a review. Front Immunol.

[57] Figueiredo JC (2022). Mission, organization, and future direction of the serological sciences network for COVID-19 (SeroNet) epidemiologic cohort studies. Open Forum Infect Dis.

[58] McClain MT (2004). The prevalence, onset, and clinical significance of antiphospholipid antibodies prior to diagnosis of systemic lupus erythematosus. Arthritis Rheum.

[59] Tovanabutra N (2022). Temporal outcomes after rituximab therapy for pemphigus vulgaris. J Invest Dermatol.

[60] Cheng SW (2002). Monitoring disease activity in pemphigus with enzyme-linked immunosorbent assay using recombinant desmogleins 1 and 3. Br J Dermatol.

