# Supplementary material for: Factors associated with immune responses to SARS-CoV-2 vaccination in individuals with autoimmune diseases
Source: JCI Insight. 2024 Jun 4;9(13):e180750. doi: 10.1172/jci.insight.180750 (PMC11383356; doi:10.1172/jci.insight.180750)
Supplement: Supplemental data [file jciinsight-9-180750-s100.pdf]

# Factors associated with immune responses to SARS-CoV-2 vaccination in autoimmune disease individuals.

## SUPPLEMENTARY METHODS, FIGURES AND TABLES

## SUPPLEMENTARY METHODS

*Clinical assessments*

*Demographic data, concomitant medications, comorbid diseases, and vaccine type were entered into a central Health Insurance Portability and Accountability Act (HIPAA)–compliant REDCap database. Disease activity was categorized as either inactive, mild, moderate, or severe using the Clinical Disease Activity Index (CDAI) for RA (1), SLEDAI-2K for SLE (2), Pemphigus Disease Area Index (PDAI) for pemphigus (3), or physician determination for other diagnoses. Disease flares were defined as an increase in disease activity prompting changes in immunosuppressive medication. Treatment details—including current use of immunosuppressive, immunomodulatory, and biologic medications; history of B cell depletion or cyclophosphamide within the last 12 months; current oral corticosteroid dose; and infusions of > 100 mg of methylprednisolone within the last 6 months—were recorded at the Pre V visit. Changes in medications were recorded at all subsequent visits. Additional medications recorded at post-vaccine visits must have been taken for > 4 weeks, except for cyclophosphamide and B cell–depleting agents, which could be taken for shorter periods. Temporary suspension of immunosuppressives or biologics at the time of vaccination according to or close to American College of Rheumatology (ACR) guidelines was also recorded (4).*

*T cell activation induced marker (AIM) assays and flow cytometry*

*Cryopreserved PBMCs were thawed and washed with AIM medium (RPMI) (Fisher, 11875135) supplemented with 5% human AB serum (Fisher, BP2525-100) and 1% penicillin-streptomycin (Fisher, 15140122). Washed PBMCs were cultured at 1 x $10^6$ cells in 200 µl AIM medium in a 96-well round bottom plate. A separate well for each donor was stimulated in 1 of 4 conditions:*

*(1) 2% $H_2O$ vehicle control (Gibco, 15230-170), (2) 2µl washed anti-CD3/CD28 beads (Thermo Scientific, 1132D), (3) 1µg/mL PepTivator CMV pp65 (Miltenyi Biotec, 130-093-435), or (4) 1µg/mL PepTivator SARS-CoV-2 Prot_S Complete (Miltenyi Biotec, 130-129-712). Anti-CD3/CD28 beads and CMV stimulations were omitted in samples with a low cell number. Stimulated PBMCs were cultured for 20 to 24 hours at 37$^o$C. Samples were analyzed in groups of 12, with each group containing 1 of 2 deidentified healthy donors (n = 37 and n = 11).*

*After incubation, PBMCs were centrifuged and washed with fluorescence-activated cell sorting (FACS) buffer (PBS/2mM EDTA/1% BSA). Cells were stained with the fluorophore conjugated antibodies and viability dye (***Supplementary Table 10***), for 30 min at 4°C. Stained PBMCs were washed twice with FACS buffer and transferred to 5 mL round bottom flow tubes. A Becton Dickerson (BD) LSR Fortessa X-20 cell analyzer was used to acquire data on all samples. Data were analyzed using BD FACS Diva (v9.0) and FlowJo (v10.8.1).*

*Autoantibody arrays*

*Antigens (***Supplementary Table 9***) were conjugated to uniquely barcoded, carboxylated magnetic beads (MagPlex-C, Luminex Corp.) as previously described (29). Five µl of bead array was added to each well of a 384-well plate (Greiner BioOne). Forty-five µl of diluted serum or plasma per well was transferred into the 384-well plate containing the bead array. Samples were incubated for 60 minutes on a shaker at room temperature and then left overnight at 4°C. Beads were then washed with 3 × 60 µl PBS-Tween on a plate washer (EL406, Biotek) and incubated with 50 µl of 1:1000 diluted R-phycoerythrin (R-PE)–conjugated, Fc-γ–specific goat anti-human IgG F(ab')2 fragment (Jackson ImmunoResearch, 106-116-098) for 30 minutes. The beads were*

*then washed with 3 × 60 µl PBS-Tween and re-suspended in 50 µl PBS-Tween, and the plates were then analyzed with a FlexMap3D™ instrument (Luminex Corp.). Beads were validated on positive control plasma or serum samples with known reactivity patterns derived from subjects with confirmed prior SARS-CoV-2 infection and/or autoimmune diseases (29). Healthy control sera were obtained by the Stanford Biobank prior to the pandemic.*

*Unbiased predictive models of anti-Spike antibody and T cell response*

*Linear regression modeling was used to determine correlations of anti-spike IgG values, CD4 T cell % AIM, or CD8 T cell % AIM at the Post V1 visit with other serological and clinical variables.*

*Preprocessing: The variables considered for inclusion in the models were: ethnicity, race, age, sex, autoimmune diagnosis, comorbid disease (diabetes, hypertension, coronary artery disease, multiple comorbidities, other, or none), BMI, disease activity severity (inactive, mild, moderate or severe), medications, months since last B cell depleting agent, total number of doses of B cell depleting agent, COVID exposure, number of weeks since full COVID-19 vaccination, vaccine type, MMF/MPA level, flare in disease activity requiring an increase or change in medication, mean corticosteroid dose since last visit, cytokine levels (IFNa1,2,6,7, IFNg, IL-17A, IL-17F, GM-CSF, TNFa, IL-6, CXCL10), anti-NC, anti-spike IgG, CD4 T cell level, and CD8 T cell level. Certain medications were considered together: B cell inhibition (ocrelizumab, obinutuzimab, ofatumumab, rituximab), TNF inhibitors (adalimumab, certolizumab, etanercept, golimumab, infliximab), IL-12/23 and IL-17A inhibitors (ustekinumab, secukinumab), Jak inhibitors and IL-6 receptor antagonist (tocilizumab), and MMF/MPA. Variables with over 50% missing data were excluded from the dataset. The log2 of the anti-spike IgG and T cell values*

*were used. KNN imputation was performed on all non-one-hot-encoded data to increase the amount of data points available for training and testing.*

*Model building: A linear regression model was used to determine correlations between the serological or clinical variables and the three outcomes of interest (anti-spike IgG values, CD4 T cell % AIM, or CD8 T cell % AIM). 70% of the data was used as a training set, of which 30% was used as a validation set. The remaining 30% of the data was used as a test set. The variables were added into the model sequentially. If including a new variable improved the performance of the linear regression model, as measured by the* $R^2$ *value, the new variable was kept in the final model. If including a new variable did not improve the model's performance, it was removed from the dataset. The final performance of the model was evaluated on a validation data set that included thirty percent of the data the testing data that was set aside prior to building the model. The model's accuracy was measured through evaluating the* $R^2$ *value. The coefficients of every variable included in the model were also reported.*

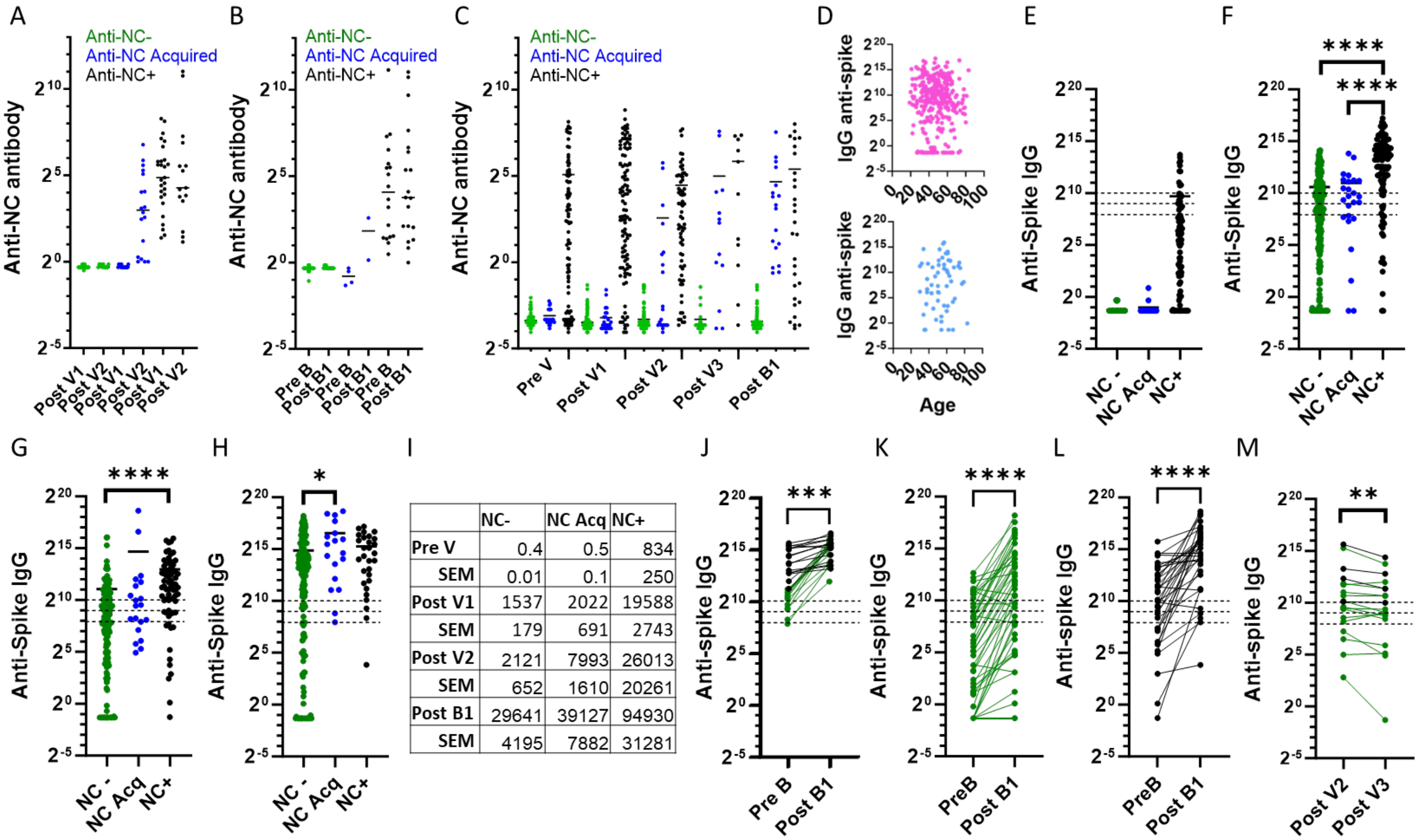


| | NC- | NC Acq | NC+ |
|---|---|---|---|
| Pre V | 0.4 | 0.5 | 834 |
| SEM | 0.01 | 0.1 | 250 |
| Post V1 | 1537 | 2022 | 19588 |
| SEM | 179 | 691 | 2743 |
| Post V2 | 2121 | 7993 | 26013 |
| SEM | 652 | 1610 | 20261 |
| Post B1 | 29641 | 39127 | 94930 |
| SEM | 4195 | 7882 | 31281 |

**Supplementary Figure 1: SARS-CoV2 Exposure Status in Autoimmune Subjects and Healthy Controls, and Relationship to Serological Response to SARS-CoV2 Vaccination.** *A, B: Anti-NC values (U/mL) in healthy controls at each visit after the initial (A) and booster (B) vaccination. C: Anti-NC values in autoimmune subjects at each visit before and after the initial vaccination, and after booster vaccination. D: Correlation of anti-spike IgG values (U/mL) with age in female (pink) and male (blue) autoimmune subjects; p=NS. E-H: Anti-spike IgG values according to anti-NC status in autoimmune subjects at the visit before the initial vaccine series (E - Pre V), at Post V1 (F), at Post V2 (G) and after booster vaccination (H - Post B1). I: Mean + SEM of anti-spike IgG values in autoimmune subjects according to anti-NC status as shown in D-G. J: Trajectory of anti-spike IgG values and after booster vaccination in anti-NC+ (black) and anti-NC- (green) healthy controls. K-L: Trajectory of anti-spike IgG values after booster vaccination (K) in anti-NC- (green) and anti-NC+ (black) autoimmune subjects. M: Trajectory of anti-spike IgG values between Post V2 and Post V3 (M) in anti-NC- (green) and anti-NC+ (black) autoimmune subjects. E-H, J-M: Dotted lines indicate IgG anti-spike values of 250, 500 and 1000. E-H: Kruskal-Wallis ANOVA with Dunn's correction for multiple comparisons. J-M: Wilcoxon matched pairs rank test; differences between anti-NC+ vs. anti-NC- subjects evaluated using Mann Whitney t test (see text). * p <0.05, *** p < 0.001, **** p < 0.0001.*

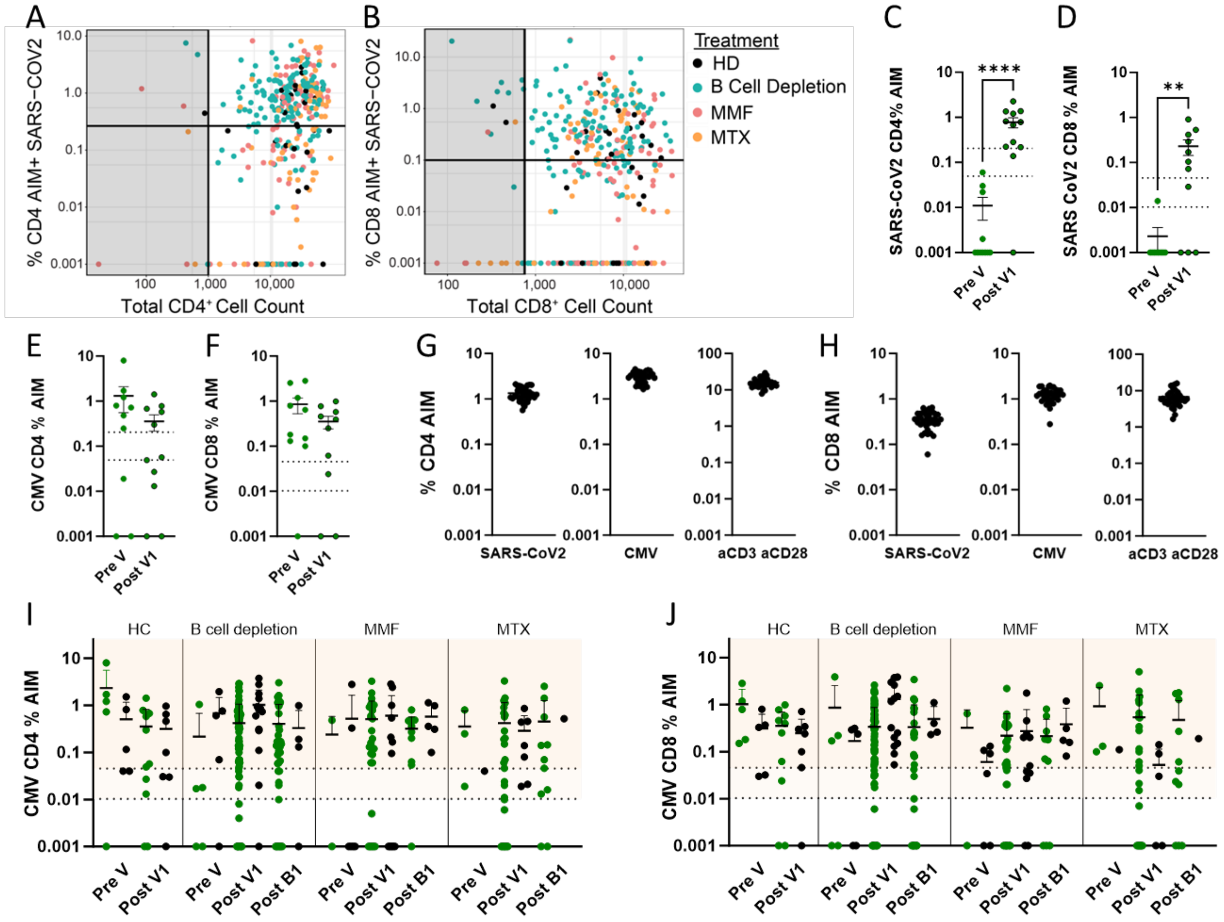


**Supplementary Figure 2. AIM Assay Quality Controls.** *A-B: Dot plot colored by treatment group comparing total CD4 (A) and CD8 (B) counts vs % AIM+ after SARS-CoV-2 peptide pool stimulation. To limit false positives/negatives, samples with fewer than 1,000 CD4 (2.85%) and 750 CD8 T (6.55%) cells were removed from subsequent analysis. C-F: CD4 (C, D) and CD8 (E, F) AIM+ frequency after SARS-CoV2 (C, D) or CMV (E, F) peptide pool stimulation of Pre V and Post V1 samples from anti-NC- healthy donors post vaccination. Positive SARS-CoV2 response thresholds, represented as dotted lines, were calculated using the uninfected Pre V group (n=11 for CD4 and n=10 for CD8) as follows: mean + 2*SD and mean + 10*SD. Statistical significance was determined by unpaired two-tailed t test. ** p < 0.01, **** p < 0.0001. G, H: CD4 (G) and CD8 (H) AIM frequencies after the indicated stimulation showing consistency of a single deidentified healthy donor included in each run of the AIM assays. Data represent mean ± SEM (n = 37). I, J: No differences in CD4 (I) and CD8 (J) AIM frequencies in response to CMV peptide pool stimulation at sequential visits according to SARS-CoV2 exposure and medication. Each data point represents an individual subject.*

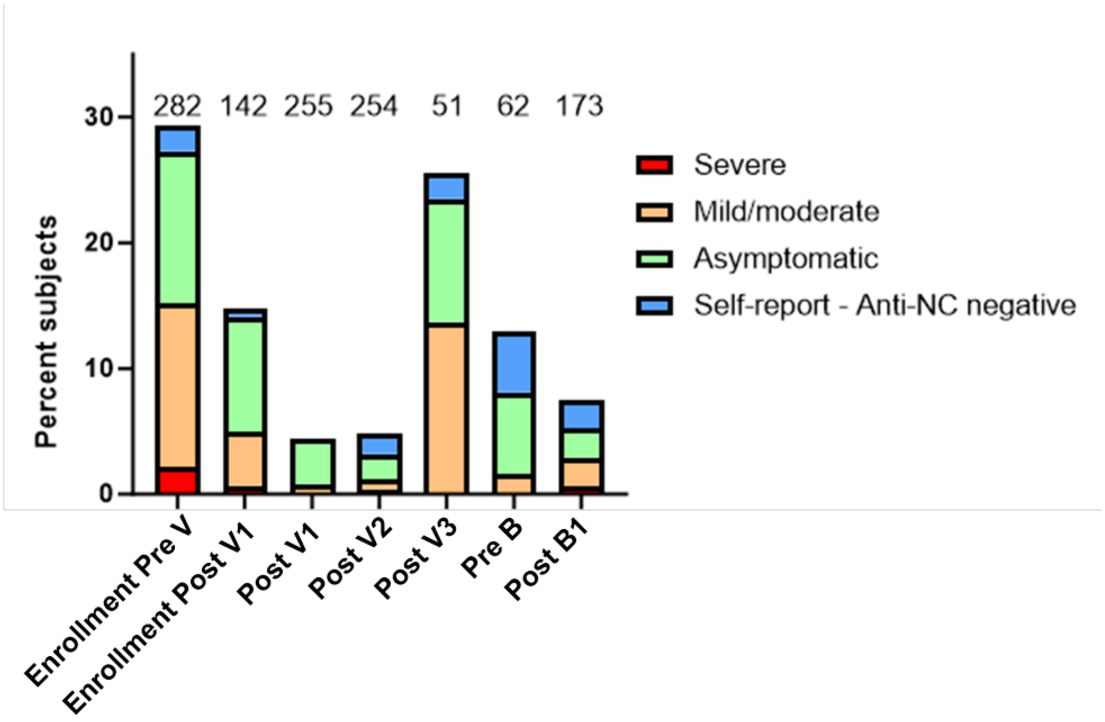


**Supplementary Figure 3.** *Breakthrough infection frequency and severity at each visit. Frequency and severity of infections acquired since the previous visit are shown in each bar as a percentage of subjects with available data at each visit. The subject number for each visit is shown above each bar.*

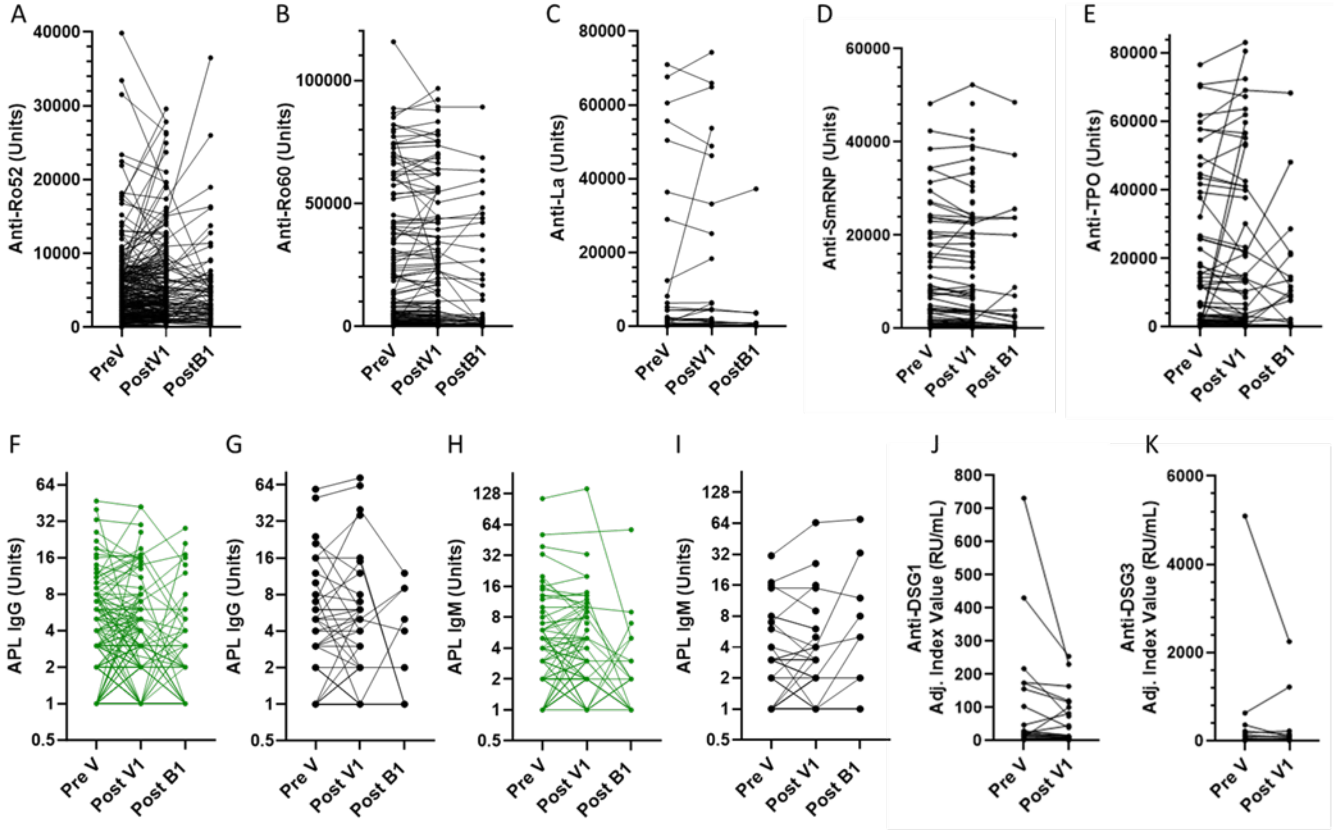


**Supplementary Figure 4: Autoantibodies to selected analytes** *A-E: Line plots showing changes in MFI levels for 5 analytes in matched samples from 241 vaccinated subjects with a range of autoimmune diseases. B-D: Autoantibodies against Ro60/SSA, La/SSB, and Smith demonstrate stable MFI levels throughout the vaccine series. E: Newly detected anti-TPO autoantibodies in a small subset of subjects. F-I: Anti-cardiolipin IgG (F, G) and IgM (H, I) autoantibodies in matched samples from 186 anti-NC- (green) and 63 anti-NC+ (black) subjects. Values >10 are considered positive. J, K: Anti-desmoglein1 (J) and 3 (K) autoantibodies in matched samples from 23 subjects with pemphigus. Each connected set of data points represents 1 subject. P = NS for all comparisons performed using Kruskal-Wallis ANOVA.*

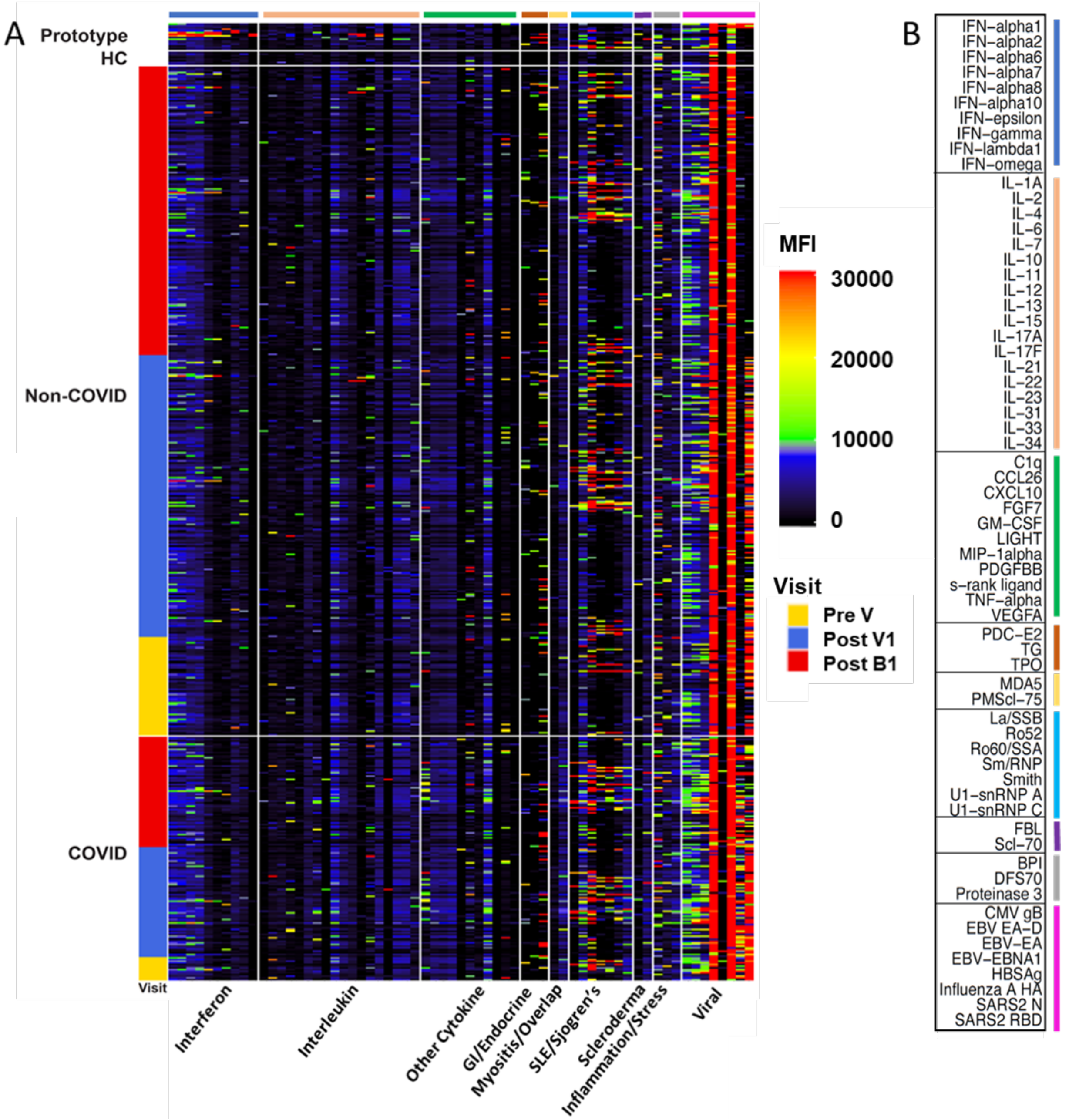


**Supplementary Figure 5. Heatmap representing serum IgG detected with an 83-plex array of cytokines and chemokines, traditional autoimmune-associated antigens, and viral antigens.** *239 vaccinated autoimmune subjects are represented, grouped by whether they were infected with SARS-CoV-2 by the Post V1 visit. Representative data from 16 prototype samples and 8 healthy control subjects are included. Within each group, samples are clustered and annotated by the visit at which the sample was taken (Pre V, yellow; Post V1, blue; Post B1, red). Analytes that were not cross-reactive and had a value above 5000 MFI are shown. Analytes in each group of antigens are color coded and individual antigens in each group of antigens are shown in B.*

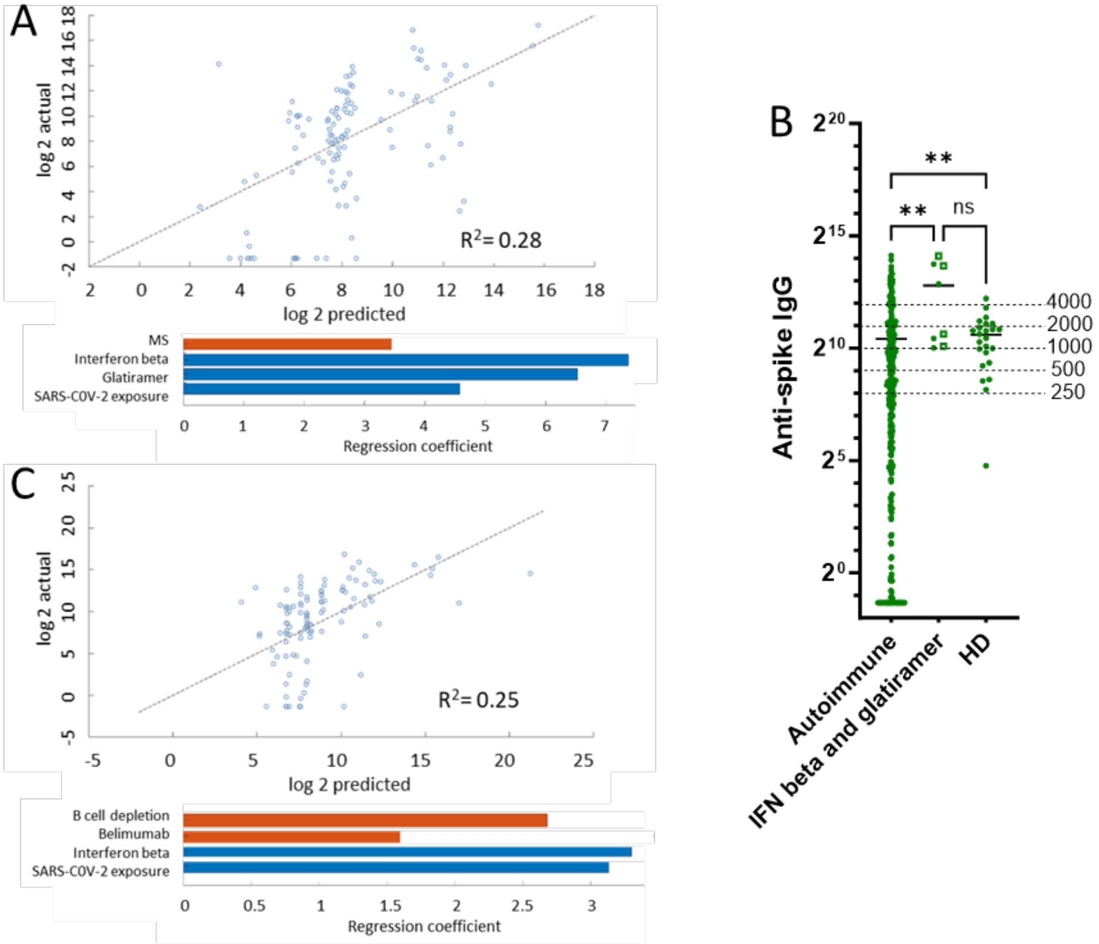


**Supplementary Figure 6. Unbiased predictive models of anti-spike IgG response**. *Models including an autoimmune diagnosis (A) or excluding an autoimmune diagnosis (C) are shown together with the top 4 variables contributing to each model. Orange bars indicate variables associated with lower anti-spike IgG values and blue bars indicate variables associated with higher anti-spike IgG values. B: Anti-spike IgG values in subjects treated with IFN beta (circles) or glatiramer (squares) are compared with those from other autoimmune subjects and healthy controls. Kruskal-Wallis ANOVA with Dunn's correction for multiple comparisons. ** $p < 0.01$.*

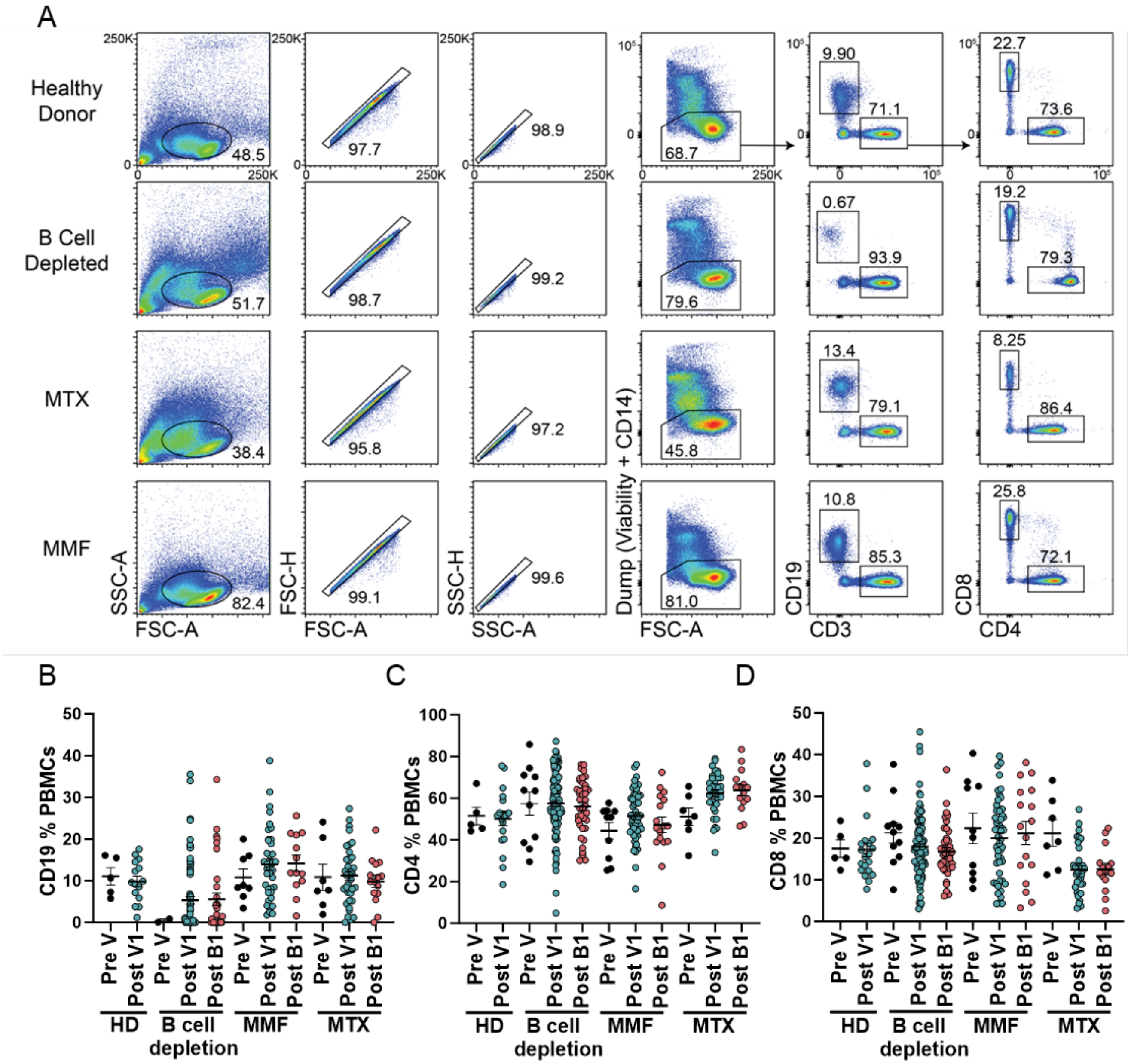


**Supplementary Figure 7. Flow Cytometry Gating and Immune Cell Frequencies.** *A: Flow gating strategy for classifying CD4+ and CD8+ cells for each treatment group and timepoint. B-D: Quantified frequency of CD19 (C), CD4 (D) and CD8 (E) populations depicted as frequency of total live lymphocytes for each treatment group. Each data point reflects a single subject. Data represent mean ± SEM. p=NS for all comparisons performed using Kruskal- Wallis ANOVA.*

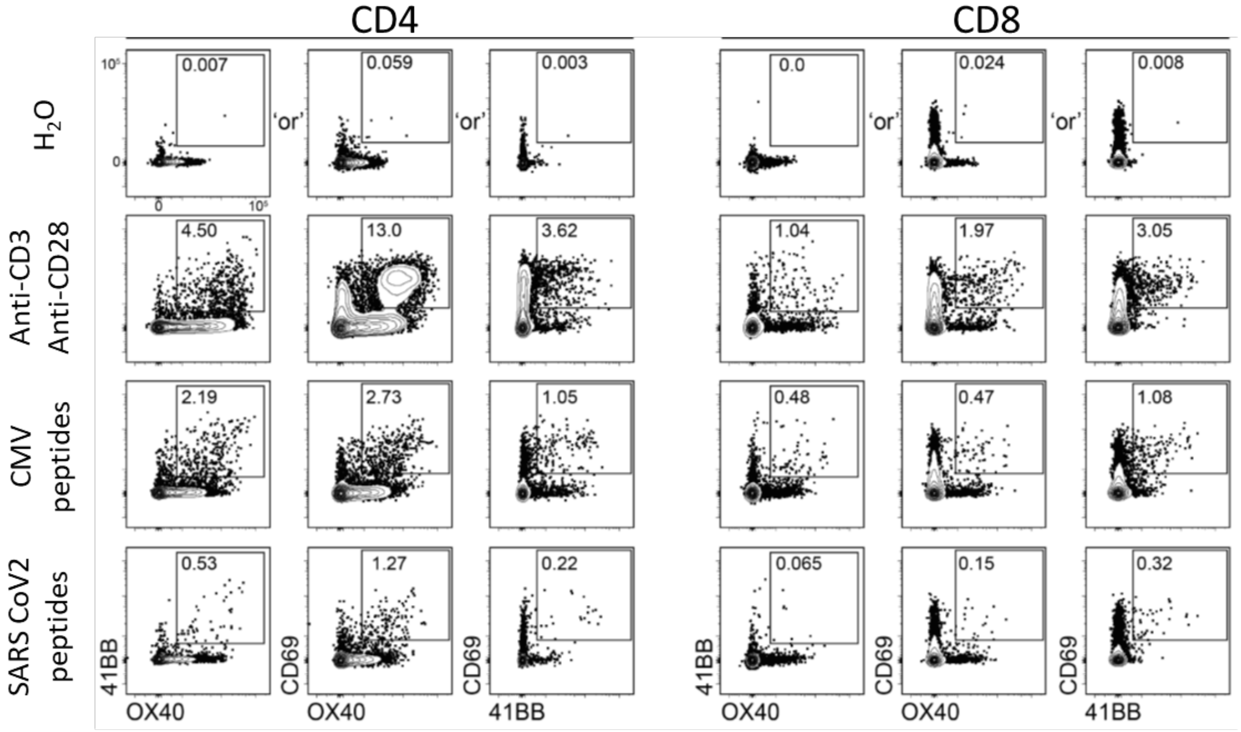


**Supplementary Figure 8. Boolean OR AIM Gating.** *Boolean logic OR flow gating strategy implemented to determine the % CD4 (A) and % CD8 (B) AIM+ frequency for each of the indicated stimulation conditions as follows: CD137+OX40+ OR CD137+CD69+ OR OX40+CD69+.*

**Supplementary Table 1: Response rates to vaccination and booster in autoimmune subjects and healthy controls**

| | Post V1 | | | | | | Post B1 | | | | |
|---|---|---|---|---|---|---|---|---|---|---|---|
| * | | **AI (397)** | **AI %** | **HC (67)** | **HC %** | * | | **AI (211)** | **AI %** | **HC (34)** | **HC %** |
| Anti-NC- (270) | <250 | 122 | 45.1 | 1/24 | 1.5 | (159) | <250 | 45 | 28.3 | 0/13 | 0 |
| | | | | | | | <4000 | 67 | 42.1 | 0/13 | 0 |
| Anti-NC acquired (25) | <250 | 8 | 32 | 0/16 | 0 | | | | | | |
| Anti-NC+ (102) | <250 | 16 | 15.7 | 0/27 | 0 | (52) | <250 | 4 | 7.7 | 0/21 | 0 |
| | <2000 | 26 | 25.5 | 0/27 | 0 | | <4000 | 14 | 27.5 | 0/21 | 0 |

AI: autoimmune subjects; HC: healthy controls

*Numbers of autoimmune subjects in each group

## Supplementary Table 2. Medication Use Associated with Anti-Spike Antibody and Anti-NC Status

| | Post V1 | | | | Post-Booster | | | |
|---|---|---|---|---|---|---|---|---|
| | Responder | | Non-Responder | | Responder | | Non-Responder | |
| **Medication** (Totals for each category in blue) | **Anti-NC-** | **Anti-NC+** | **Anti-NC-** | **Anti-NC+** | **Anti-NC-** | **Anti-NC+** | **Anti-NC-** | **Anti-NC+** |
| **B cell depletion alone** | **24** | **2** | **45** | **6** | **23** | **2** | **36** | **3** |
| rituximab (RTX) | 19[5] | 2 | 15 | 2 | 15 | 2 | 11 | 2 |
| ocrelizumab (Ocr) | 5 | - | 29 | 4 | 7 | - | 23 | 1 |
| obinutuzumab | - | - | 1 | - | 1 | - | 1 | - |
| ofatumumab | - | - | - | - | - | - | 1 | - |
| **B cell deletion + other drug** | **2** | **3** | **9** | **5** | **4** | **0** | **0** | **1** |
| RTX + HCQ | - | 1 | 2 | 1 | 1 | - | - | - |
| RTX + MMF | - | - | 1 | - | - | - | - | - |
| RTX + MMF + HCQ | 2[2] | 1[18] | 1[19] | 1 | 3[ff] | - | - | - |
| RTX + MMF + other | - | - | - | 1[q] | - | - | - | - |
| RTX + azathioprine | - | 1[6] | - | - | - | - | - | - |
| RTX + azathioprine + HCQ | - | - | - | 1 | - | - | - | - |
| RTX + methotrexate | - | - | 1 | - | - | - | 2 | - |
| RTX + other | - | - | 4[m] | 1 | - | 1 | 4[nn] | - |
| RTX + other + HCQ | - | - | - | - | - | - | - | - |
| Ocr + methotrexate (MTX) | - | - | - | - | - | - | - | 1 |
| **Mycophenolic acid or mycophenolate mofetil** | **23** | **21** | **33** | **0** | **19** | **8** | **8** | 0 |
| Mycophenolic acid (MPA) | | | | | | | | |
| alone | - | - | 1 | - | 2[23] | - | - | - |
| + HCQ | - | 1[4] | - | - | - | - | - | - |
| + belimumab | - | - | - | - | 1[cc] | - | - | - |
| + other | - | - | 1[ll] | - | 1[ll] | - | - | - |
| Mycophenolate mofetil (MMF) | | | | | | | | |
| alone | 5[10] | 6[17] | 6[1] | - | 3 | 2 | 2[25] | - |
| + HCQ | 14[16] | 13[15] | 12[14] | - | 9[21] | 6[20] | 2 | - |
| + belimumab | - | - | 3 | - | 1[kk] | - | 2[21] | - |
| + belimumab + HCQ | 1 | - | 4 | - | - | - | 2 | - |
| + belimumab + methotrexate + HCQ | - | - | 1 | - | - | - | - | - |
| + belimumab + other | - | - | 1 | - | - | - | - | - |
| + methotrexate + HCQ | - | | 1[3] | - | - | - | - | - |
| + other | 1[a] | - | - | - | - | - | - | - |
| + other + HCQ | 2[o] | 1[i] | 3[j] | - | 2[ee] | - | - | - |
| **Methotrexate (MTX)** | **27** | **9** | **13** | **0** | **11** | **4** | **5** | **0** |

| | | | | | | | | |
|---|---|---|---|---|---|---|---|---|
| alone | 13[7] | 1 | 6[9] | - | 5[22] | 2 | 2 | - |
| + HCQ | 3[8] | 1 | 2[13] | - | 2[dd] | - | 1 | - |
| + TNFi | 7[d] | 4[h] | 2[r] | - | 1 | 1[r] | 2[hh] | - |
| + TNFi + HCQ | 1[r] | - | - | - | - | 1[r] | - | - |
| + belimumab | - | - | 1[12] | - | - | - | - | - |
| + belimumab + HCQ | 1[11] | 1 | - | - | - | - | - | - |
| + ocrelizumab | - | 1 | - | - | - | - | - | - |
| + other | 2[e] | 1[i] | 2[f] | - | 3[aa] | - | - | - |
| **Belimumab** | **4** | **2** | **7** | **0** | **5** | **0** | **0** | **1** |
| alone | 1 | - | 1 | - | - | - | - | 1[24] |
| + HCQ | 3 | 2 | 5 | - | 5 | - | - | - |
| + azathioprine + HCQ | - | - | 1 | - | - | - | - | - |
| **TNF inhibitors** | **5** | **2** | **3** | **2** | **2** | **1** | **1** | **0** |
| alone | 4[u] | 2[r] | 1[q] | 1[t] | 2[gg] | 1[t] | - | - |
| + HCQ | 1[w] | - | - | - | - | - | - | - |
| + other | - | - | 1[r] | - | - | - | 1[y] | - |
| **Azathioprine (AZA)** | **7** | **4** | **5** | | **5** | **6** | **0** | **1** |
| alone | 3 | 2 | 2 | - | 2 | 2 | - | - |
| + HCQ | 4 | 1 | 1 | - | 2 | 1 | - | 1[pp] |
| + HCQ + other | - | 1[i] | 2[p] | - | 1[ii] | 1[c] | - | - |
| **HCQ** | **38** | **19** | **7** | **5** | **11** | **6** | **1** | **1** |
| alone | 36 | 18* | 6 | 5 | 11[jj] | 6 | 1 | 1 |
| + other | 2[s] | 1 | 1[y] | - | - | - | - | - |
| **Other** | **18** | **8** | **8** | **3** | **13** | **5** | **15** | **3** |
| alone | 18[l] | 8[k] | 8[v] | 2[f] | 13[bb] | 5[b] | 15[oo] | 3[mm] |
| + abatacept | - | - | - | 1[z] | - | - | - | - |
| **None** | **14** | **5** | **2** | **0** | **3** | **0** | **1** | **0** |
| **TOTAL** | **162** | **75** | **131** | **20** | **96** | **31** | **73** | **10** |

a. voclosporin (1)

b. tacrolimus (1), interferon beta (1), secukinumab (1), tocilizumab (1), fingolimod (1)

c. clinical trial drug (1)

d. adalimumab (5), golimumab (1), etanercept (1)

e. JAK inhibitor (1), ustekinumab (1); 1 subject in which methotrexate was held and ustekinumab was continued (R131); 1 subject in which methotrexate was held and JAK inhibitor continued (R023)

f. abatacept; 1 subject in which both methotrexate and abatacept was held (R011); 1 subject in which abatacept was held (R107)

g. golimumab (1)

h. certolizumab (1), etanercept (3); 1 subject with both methotrexate and etanercept held for vaccine dosing (F701); 1 subject in which methotrexate was stopped but etanercept continued (R001)

i. clinical trial drug (baricitinib) – this subject was non-adherent to MMF (B051)

j. tacrolimus (2), other (1)

k. fingolimod (1), glatiramer acetate (1), interferon beta (1), secukinumab (1), tocilizumab (1), cladribine (1), JAK inhibitor (1), other (1)

l. dimethyl fumarate (1), glatiramer acetate (3), interferon beta (5), natalizumab (1), other (9)

m. cyclophosphamide IVSS (1), other (3)

n. other (1)

o. IVIG (1), other (1); 1 subject who was on “other medication” (colchicine) was non-adherent to MMF (F457)

p. abatacept (1), cyclophosphamide IVSS (1); 1 subject in which abatacept was held and both azathioprine and hydroxychloroquine were continued (R093)

q. golimumab

r. adalimumab; 1 subject in which methotrexate was stopped and adalimumab was held at Post V1 (R008); 1 subject in which methotrexate was held and adalimumab and hydroxychloroquine were continued at Post V1 (F691)

s. dapsone

t. infliximab

u. adalimumab (3), infliximab (1)

v. fingolimod (5), tacrolimus (1), cyclosporine (1), other (1)

w. certolizumab

x. IVIG

y. leflunomide

z. leflunomide + abatacept (abatacept held for vaccine dosing)

aa. abatacept (1), ustekinumab (1), 1 subject in which JAK inhibitor started at Post V2 and adalimumab stopped at Post V2

bb. glatiramer acetate (4), interferon beta (3), mepolizumab (1), leflunomide (2), teriflunomide (1), natalizumab (1); 1 subject for which abatacept was stopped prior to booster dosing

cc. 1 subject in which mycophenolic acid was started at Post V1 and then held for booster dosing

dd. 1 subject in which both methotrexate and HCQ were held for booster dosing

ee. 1 subject non-adherent to MMF, and clinical trial drug started at Post V3; 1 subject non-adherent to MMF and on tacrolimus

ff. 1 subject non-adherent to MMF, and received a mean steroid dose of 400 mg prednisone equivalent between Pre V and Post V1

gg. adalimumab (1), infliximab (1)

hh. adalimumab (2), 1 of these subjects had been on belimumab at Post V1 that was stopped, and adalimumab was started

ii. cyclophosphamide IVSS (1)

jj. 1 subject in which HCQ was held for booster dosing; 1 subject who had also been on belimumab that was stopped at Post V2

kk. 1 subject in which MMF was held for booster dosing, belimumab was started between the Pre B and Post B1 visit, and was on prednisone 60 mg daily at the time of booster dosing

ll. tacrolimus

mm. fingolimod (2), cladribine (1)

nn. eculizumab (1), leflunomide (1); 1 subject with apremilast started at Post V1; 1 subject also received cyclophosphamide IVSS

oo. fingolimod (11), interferon beta (2), glatiramer acetate (1), tocilizumab (1)

pp. subject had also been on belimumab that was stopped at Post V2

1. 1 subject was MMF non-adherent and on an equivalent dose of prednisone ≥ 30 mg; 1 subject in which MMF was held (F694)
2. 1 subject on an equivalent dose of prednisone ≥ 30 mg, and was non-adherent to MMF (F323)
3. 1 subject in which MMF and hydroxychloroquine were stopped, and methotrexate continued, also on an equivalent dose of prednisone ≥ 30 mg (500497)
4. subject on an equivalent dose of prednisone ≥ 30 mg
5. 1 subject on an equivalent dose of prednisone ≥ 30 mg
6. azathioprine held for vaccine dosing
7. 3 subjects in which methotrexate was held (R118, F677, R038)
8. 1 subject in which methotrexate was held and hydroxychloroquine was continued (R105)

9. 1 subject in which methotrexate was held (F717)
10. 2 subjects in which MMF was held (R148, ACE-P-012)
11. 1 subject in which belimumab was held, and methotrexate and hydroxychloroquine were continued (F158)
12. belimumab was stopped and adalimumab started and taken between the 1st and 2nd Pfizer vaccines, while methotrexate was continued (500940)
13. 1 subject in which methotrexate was held and hydroxychloroquine was continued (F355)
14. 1 subject in which MMF was held and hydroxychloroquine was continued (F692)
15. 5 subjects were non-adherent to MMF
16. 3 subjects were non-adherent to MMF
17. 1 subject was non-adherent to MMF (E3588)
18. subject was non-adherent to MMF (B028)
19. 1 subject was non-adherent to MMF (E3702)
20. 2 subjects non-adherent to MMF
21. 1 subject non-adherent to MMF
22. 2 subjects in which MTX was held for booster dosing
23. 1 subject in which MPA held for booster dosing
24. 1 subject on prednisone 30 daily at the time of booster dosing
25. 1 subject in which MMF was held for booster dosing

*1 subject who was ANA positive, without a clear autoimmune diagnosis, and was started on hydroxychloroquine before the Post V1 visit

** 9 subjects who received B cell depletion alone, with the last dose > 60 months prior to vaccination, were excluded from this table

**Supplementary Table 3: Medication Use Associated with Anti-Spike Antibody Response in Subjects with both a Post V1 and a Post B1 Visit**

| | Double Non-Responder (n=36) | | Single Non-Responder (n=41) | | Responder (n=76) | |
|---|---|---|---|---|---|---|
| **Medication** (Totals for each category in blue) | **Anti-NC-** | **Anti-NC+** | **Anti-NC-** | **Anti-NC+** | **Anti-NC-** | **Anti-NC+** |
| **B cell depletion alone** | **18** | **2** | **9** | **1** | **13** | **-** |
| rituximab (RTX) | 4 | 1 | 4 | 1 | 11 | - |
| ocrelizumab (Ocr) | 13[1] | 1 | 5 | - | 2 | - |
| obinutuzumab | 1 | - | - | - | - | - |
| **B cell depletion + other drug** | **4** | **2** | **1** | **1** | **3** | **0** |
| RTX + MMF + HCQ | - | - | - | - | 2[!!!] | - |
| RTX + belimumab + MMF + HCQ | - | 1[⊥] | 1[##] | - | - | - |
| RTX + cyclophosphamide IVSS | 1[1] | - | - | - | - | - |
| RTX + eculizumab | 1 | - | - | - | - | - |
| RTX + methotrexate | 1 | - | - | - | - | - |
| RTX + leflunomide | - | - | - | - | 1 | - |
| RTX + leflunomide + apremilast | 1 | - | - | - | - | - |
| RTX + IVIG | - | - | - | 1[$$] | - | - |
| Ocr + methotrexate (MTX) | - | 1 | - | - | - | - |
| **Mycophenolic acid or mycophenolate mofetil** | **4** | **1** | **9** | **2** | **8** | **6** |
| Mycophenolic acid (MPA) | | | | | | |
| alone | - | - | 1 | - | - | - |
| + tacrolimus | - | - | 1 | - | - | - |
| Mycophenolate mofetil (MMF) | | | | | | |
| alone | 1 | - | - | - | 4[*] | 1 |
| + HCQ | - | - | 4[#,1] | 1[$$] | 4[***] | 5** |
| + belimumab | 2[1] | - | - | - | - | - |
| + belimumab + HCQ | 1 | - | 2[@] | - | - | - |
| + belimumab + MPA | - | - | 1 | - | - | - |
| + tacrolimus + HCQ | - | - | - | 1 | - | - |
| **Belimumab** | **0** | **1** | **2** | **0** | **3** | **0** |
| + HCQ | - | - | 2 | - | 3[+] | - |
| + azathioprine + HCQ | - | 1[!!] | - | - | - | - |
| **Methotrexate (MTX)** | **0** | **0** | **7** | **1** | **6** | **2** |
| alone | - | - | 4 | - | 2[&] | - |
| + HCQ | - | - | 1[@@] | 1[⊥⊥] | 1 | - |
| + abatacept | - | - | 1[$] | - | - | - |
| + adalimumab | - | - | - | - | 2 | 1[$$] |
| + adalimumab + HCQ | | - | - | - | - | 1[&&] |

| | | | | | | |
|---|---|---|---|---|---|---|
| + ustekinumab | - | - | - | - | 1 | - |
| + adalimumab + Jak inhibitor | - | - | 1[!] | - | - | - |
| **TNFi** | **1** | **0** | **0** | **0** | **2** | **1** |
| infliximab | - | - | - | - | 1 | 1[$$] |
| + sulfasalazine | - | - | - | - | 1[^^] | - |
| + leflunomide | 1 | - | - | - | - | - |
| **Azathioprine (AZA)** | **0** | **0** | **3** | **0** | **2** | **2** |
| alone | - | - | 1 | - | 1 | 1 |
| + HCQ | - | - | 1 | - | 1 | - |
| + cyclophosphamide IVSS + HCQ | - | - | 1[1] | - | - | - |
| + HCQ + other | - | - | - | - | - | 1[&&&] |
| **Hydroxychloroquine alone** | - | - | 1 | 1 | 9[++] | 7 |
| **Cladribine** | - | - | - | - | - | 1 |
| **Fingolimod** | 4 | - | 1 | - | - | - |
| **Glatiramer acetate** | - | - | - | - | 4 | - |
| **Interferon-beta** | - | - | - | - | 5 | 1 |
| **Mepolizumab** | - | - | 1 | - | - | - |
| **Natalizumab** | - | - | - | - | 1 | - |
| **Tacrolimus alone** | - | - | - | 1[$$] | - | - |
| **TOTAL** | **31** | **5** | **34** | **7** | **56** | **20** |

# 1 subject non-adherent to MMF at the Post V1 visit

⊥ 1 subject was on 30 mg of prednisone at the Pre V visit

* 2 subjects on MMF only at Post B1, not Post V1

@ 1 subject only on MMF at Post V1, and only on belimumab at Post B1 (MMF held); this subject was also on 60 mg of prednisone at Post V1

$ MTX and abatacept held at Post V1

! Jak-inhibitor added at Post V2; adalimumab stopped at Post V2

& 1 subject held MTX at booster

## only on belimumab at Post V1; on rituximab and MMF at Post B1; this subject was also on 30 mg of prednisone at the Pre V visit

^ 1 subject on belimumab only at Post V1, not at Post B1 (stopped)

** 2 subjects non-adherent to MMF at the Post V1 visit, 1 of these subjects also had a breakthrough infection

!! belimumab held for booster dosing; HCQ and azathioprine continued; also had breakthrough infection

@@ MTX held at Post V1 and at booster; HCQ continued at Post V1 and held at booster

&& MTX held at Post V1; subject also with breakthrough infection

^^ on adalimumab only at the Post V1 visit

&&& clinical trial drug

+ belimumab stopped at Post V2

++ 1 subject with HCQ held at booster visit

*** 3 subjects non-adherent to MMF at the Post V1 visit

!!! 1 subject non-adherent to MMF at the Post V1 visit; also received a mean of 400 mg prednisone between the Pre V and Post V1 visits

⊥⊥ 1 subject on HCQ alone at the Post V1 visit, and was on HCQ and methotrexate at the Post B1 visit

1. 1 subject with breakthrough infection, anti-NC negative

$$ also, with breakthrough infection

**Supplementary Table 4: Medications Held/Stopped According to Anti-Spike Antibody and Anti-NC Status**

| | Post V1 | | | | Post-Booster | | | |
|---|---|---|---|---|---|---|---|---|
| | Responder | | Non-Responder | | Responder | | Non-Responder | |
| | Anti-NC- | Anti-NC+ | Anti-NC- | Anti-NC+ | Anti-NC- | Anti-NC+ | Anti-NC- | Anti-NC+ |
| **Rituximab + other drug** | | | | | | | | |
| + azathioprine | | AZA held | | | | | | |
| **Mycophenolic acid (MPA)** | | | | | | | | |
| alone | | | | | MPA held | | | |
| + belimumab | | | | | MPA held | | | |
| **Mycophenolate mofetil (MMF)** | | | | | | | | |
| alone | MMF held (2) | | MMF held | | | | MMF held | |
| + HCQ | | | MMF held | | MTX and HCQ held | | | |
| + belimumab | | | | | MMF held | | | |
| + methotrexate + HCQ | | | MMF and HCQ stopped | | | | | |
| **Methotrexate (MTX)** | | | | | | | | |
| alone | MTX held (3) | | MTX held | | MTX held (2) | | | |
| + HCQ | MTX held (1) | | MTX held | | | | | |
| + TNFi | | MTX and etanercept held (1); MTX stopped but etanercept continued | | | | MTX stopped and adalimumab held | | |
| + TNFi + HCQ | | | | | | MTX held | | |
| + belimumab | | | belimumab stopped | | | | | |
| + belimumab + HCQ | belimumab held | | | | | | | |
| + other | MTX held (2) | | MTX and abatacept held | | | | | |
| **Azathioprine (AZA)** | | | | | | | | |
| + HCQ + other | | | abatacept held | | | | | |
| **HCQ** | | | | | | | | |
| alone | | | | | HCQ held | | | |
| **Other** | | | | | | | | |
| alone | | | | abatacept held | abatacept stopped | | | |

| | | | | | | | | |
|---|---|---|---|---|---|---|---|---|
| + abatacept | | | | abatacept held | | | | |
| **SUMMARY** | MTX held (6); MMF held (2); belimumab held (1) | MTX and etanercept held (1), MTX stopped (1); AZA held (1) | MTX held (2); MTX and abatacept held (1); MMF held (2); MMF and HCQ stopped (1); belimumab stopped (1); abatacept held (1) | abatacept held (2) | MTX held (2), MTX and HCQ held (1); MPA held (2); MMF held (1); HCQ held (1); abatacept stopped (1) | MTX stopped and adalimumab held (1); MTX held (1) | MMF held (1) | none |

**Supplementary Table 5: Names of other drugs (besides B cell depletion, MMF or MPA, or methotrexate) taken by subjects who had T cell assays performed**

| Medications | Number of Subjects |
|---|---|
| azathioprine, cyclophosphamide, hydroxychloroquine | 1 |
| azathioprine, belimumab, hydroxychloroquine | 1 |
| azathioprine alone | 6 |
| azathioprine, hydroxychloroquine | 8 |
| belimumab, hydroxychloroquine | 9 |
| belimumab alone | 1 |
| adalimumab | 1 |
| adalimumab | 2 |
| fingolimod | 1 |
| golimumab | 1 |
| infliximab | 1 |
| leflunomide | 1 |
| mepolizumab | 1 |
| hydroxychloroquine alone | 2 |
| etanercept | 1 |
| interferon-beta | 1 |
| glatiramer acetate | 1 |
| **TOTAL** | 39 |

**Supplementary Table 6: Vaccine Received, Disease Diagnosis, and Flare Severity**

| Diagnosis | Visit | Flare Severity | Vaccine Received |
|---|---|---|---|
| Inflammatory Myositis | Post V1 | Moderate | Johnson and Johnson |
| Vasculitis | Post V1 | Moderate | Pfizer |
| Rheumatoid Arthritis | Post V1 | Mild | Pfizer |
| Rheumatoid Arthritis | Post V1 | Moderate | Pfizer |
| Inflammatory Myositis | Post V1 | Moderate | Moderna |
| Scleroderma, Interstitial Lung Disease | Post V1 | Severe | Pfizer |
| Rheumatoid Arthritis | Post V1 | Mild | Pfizer |
| Mixed Connective Tissue Disease | Post V1 | Severe | Pfizer |
| Eosinophilic Granulomatosis with Polyangiitis (EGPA) | Post V1 | Mild | Pfizer |
| Systemic Lupus Erythematosus | Post V1 | Mild | Pfizer |
| Rheumatoid Arthritis | Post V1 | Moderate | Pfizer |
| Rheumatoid Arthritis | Post V1 | Moderate | Johnson and Johnson |
| Pemphigus Foliaceus | Post V1 | Mild | Pfizer |
| Systemic Lupus Erythematosus | Post V1 | Moderate | Johnson and Johnson |
| Systemic Lupus Erythematosus | Post V1 | Moderate | Pfizer |
| Systemic Lupus Erythematosus | Post V1 | Moderate | Pfizer |
| Systemic Lupus Erythematosus | Post B1 | Mild | Unknown |
| Systemic Lupus Erythematosus | Post B1 | Mild | Pfizer |
| Pemphigus Vulgaris | Post B1 | Moderate | Moderna |
| Pemphigus Vulgaris | Post B1 | Moderate | Pfizer |
| Systemic Lupus Erythematosus | Post B1 | Severe | Moderna |
| Systemic Lupus Erythematosus | Post B1 | Moderate | Pfizer |
| Systemic Lupus Erythematosus | Post B1 | Moderate | Pfizer |
| Systemic Lupus Erythematosus | Post B1 | Moderate | Pfizer |
| Systemic Lupus Erythematosus | Post B1 | Severe | Moderna |
| Rheumatoid Arthritis | Post B1 | Severe | Moderna |
| Rheumatoid Arthritis | Post B1 | Mild | Pfizer |
| Rheumatoid Arthritis | Post B1 | Moderate | Pfizer |
| Rheumatoid Arthritis | Post B1 | Moderate | Moderna |
| Psoriatic Arthritis | Post B1 | Moderate | Pfizer |
| Sjogren’s Syndrome | Post B1 | Mild | Pfizer |
| Inflammatory Myositis | Post B1 | Moderate | Pfizer |
| Ulcerative Colitis/Enteropathic Colitis | Post B1 | Mild | Pfizer |
| Rheumatoid Arthritis | Post B1 | Mild | Moderna |
| Systemic Lupus Erythematosus | Post B1 | Mild | Moderna |

**Supplementary Table 7: Flares**

| Post V1 Vaccine Type | Subjects with Flare (n=16) | Subjects without Flare (n=235) | p |
|---|---|---|---|
| Pfizer | 12 (75%) | 160 (68%) | p=NS |
| Moderna | 1 (6%) | 60 (25%) | |
| Johnson and Johnson | 3 (19%) | 11 (5%) | |
| Unknown | 0 | 4 (2%) | |
| **Post B1 Vaccine Type** | **Subjects with Flare (n=19)** | **Subjects without Flare (n=187)** | **p** |
| Pfizer | 11 (58%) | 105 (56%) | p=NS |
| Moderna | 7 (37%) | 66 (35%) | |
| Johnson and Johnson | 0 | 3 (2%) | |
| Unknown | 1 (5%) | 13 (7%) | |

**Supplementary Table 8: Breakthrough infections**

| Visit | Medication 1 | Medication 2 | Diagnosis | Infection | Anti-NC | Anti-Spike IgG at prior visit |
|---|---|---|---|---|---|---|
| Post V2 | ocrelizumab | | MS | Symptomatic | Neg | 152 |
| Post V2 | MTX | abatacept | RA | Symptomatic | Neg | 46.8 |
| Post V2 | RTX | CTX | ANCA+ Vasculitis | Symptomatic | Neg | 1.24 |
| Post V2 | MMF | HCQ | SLE | Asymptomatic | Pos | 158 |
| Post V2 | HCQ | | SLE | Symptomatic | Pos | 2205 |
| Post V2 | MTX | | RA | Asymptomatic | Pos | 445 |
| Post V2 | HCQ | prednisone 30mg | SLE | Symptomatic | Pos | 839 |
| Post V2 | RTX | | RA | Hospitalized | Pos | 0.4 |
| Post V2 | RTX | IVIg | CVID | Asymptomatic | Pos | 188 |
| Post V2 | azathioprine | belimumab | SLE | Symptomatic | Pos | 0.4 |
| Post V2 | IVIg | | SLE | Asymptomatic | Pos | 3680 |
| Post V2 | RTX | MMF | RA | Symptomatic | Pos | 2.2 |
| Post V3 | MMF | HCQ | SLE | Symptomatic | Neg | 343 |
| Post V3 | RTX | | Pemphigus | Symptomatic | Pos | 39.7 |
| Post V3 | MMF | HCQ | SLE | Asymptomatic | Pos | 5220 |
| Post V3 | HCQ | | SLE | Symptomatic | Pos | 241 |
| Post V3 | MMF | voclosporin | SLE | Asymptomatic | Pos | 4150 |
| Post V3 | Dapsone | HCQ | SLE | Symptomatic | Pos | 162 |
| Post V3 | adalimumab | | PsA | Asymptomatic | Pos | 824 |
| Post V3 | MTX | | RA | Asymptomatic | Pos | 1410 |
| Post V3 | azathioprine | | Scleroderma/ILD | Symptomatic | Pos | 652 |
| Post V3 | belimumab | | SLE | Symptomatic | Pos | 2655 |
| Post V3 | None | | SLE | Asymptomatic | Pos | 1030 |
| Post V3 | MMF* | | SLE | Symptomatic | Pos | 11930 |
| Post V3 | None | | SLE | Symptomatic | Pos | *N/A |
| Pre B | ocrelizumab | | MS | Symptomatic | Neg | N/A |
| Pre B | RTX | | MS | Symptomatic | Neg | N/A |
| Pre B | RTX | | Pemphigus | Symptomatic | Neg | N/A |
| Pre B | HCQ | | SLE | Asymptomatic | Pos | 553 |
| Pre B | azathioprine | | SLE | Symptomatic | Pos | N/A |
| Pre B | fingolimod | | MS | Asymptomatic | Pos | N/A |
| Pre B | RTX | | Pemphigus | Asymptomatic | Pos | N/A |
| Pre B | HCQ | | SLE | Asymptomatic | Pos | N/A |
| Post B1 | ocrelizumab | | MS | Symptomatic | Neg | 18.3 |
| Post B1 | azathioprine (previous CTX) | HCQ | SLE | Symptomatic | Neg | 26.3 |

| | | | | | | |
|---|---|---|---|---|---|---|
| Post B1 | MMF | belimumab | SLE | Symptomatic | Neg | 1.09 |
| Post B1 | MMF | | SLE | Symptomatic | Neg | 96.4 |
| Post B1 | RTX | leflunomide | RA | Hospitalized | Neg | 0.45 |
| Post B1 | HCQ | | SLE | Asymptomatic | Pos | 744 |
| Post B1 | azathioprine | HCQ | SLE | Symptomatic | Pos | 301 |
| Post B1 | MTX | adalimumab | RA | Symptomatic | Pos | 4070 |
| Post B1 | MTX | adalimumab | RA | Asymptomatic | Pos | 1120 |
| Post B1 | MTX | | RA | Asymptomatic | Pos | 1830 |
| Post B1 | azathioprine | | SLE | Asymptomatic | Pos | 55.3 |
| Post B1 | tacrolimus | | SLE | Symptomatic | Pos | 139 |
| Post B1 | MMF | | Inflammatory Myositis | Symptomatic | Pos | 696 |
| Post B1 | MTX | | PsA | Asymptomatic | Pos | 522 |
| Post B1 | infliximab | | IBD | Symptomatic | Pos | 553 |
| Post B1 | MMF | HCQ | SLE | Symptomatic | Pos | N/A |
| Post B1 | MMF* | | SLE | Symptomatic | Pos | 8780 |
| Post B1 | MMF | | MCTD | Symptomatic | Pos | N/A |
| Post B1 | HCQ | | SLE | Symptomatic | Pos | 109 |

**  MMF level = undetectable at Post V1*

***N/A Not available*

**Supplementary Table 9: Analytes used on autoantibody arrays**

| Bead ID | Antigen | Vendor | Catalog # |
|---|---|---|---|
| **Control Analytes** | | | |
| 1 | Bare Bead | | |
| 2 | Human IgG from serum | Sigma | I4506 |
| 4 | Anti-Human IgG Fc fragment specific | Jackson | 109-005-008 |
| 7 | Anti-Human IgG (H+L) | Jackson | 109-005-003 |
| 8 | Anti-Human IgG F(ab') fragment specific | Jackson | 109-005-006 |
| **Cytokines** | | | |
| 9 | IFN-alpha1 | Prospec | CYT-291 |
| 12 | IFN-alpha2 | R&D | 11101-2 |
| 13 | IFN-alpha6 | Origene | TP760329 |
| 14 | IFN-alpha7 | Prospec | CYT-196 |
| 15 | IFN-alpha8 | Sino Biological | 10347-H08H |
| 18 | IFN-alpha10 | Sino Biological | 10349-H08H |
| 19 | IFN-epsilon | R&D | 9667-ME-025/CF |
| 20 | IFN-gamma | Peprotech | 300-02 |
| 21 | IFN-lambda1 | Peprotech | 300-02L |
| 22 | IFN-lambda2 | Peprotech | 300-02K |
| 25 | IFN-omega | Peprotech | 11395-1 |
| 26 | IL-10 | Peprotech | 200-10 |
| 28 | IL-11 | Prospec | CYT-214 |
| 29 | IL-12 | Sigma | SRP3073-10UG |
| 30 | IL-13 | Peprotech | 200-13 |
| 31 | C3a | R&D | 3677-C3-025 |
| 32 | IL-15 | Peprotech | 200-15 |
| 33 | IL-17A | Peprotech | 200-17 |

| | | | |
|---|---|---|---|
| 34 | IL-17F | Peprotech | 200-25 |
| 35 | IL-1A | Peprotech | 200-01A |
| 36 | IL-1RA | Peprotech | 200-01RA |
| 38 | IL-2 | Peprotech | 200-02 |
| 39 | IL-4 | Peprotech | 200-04 |
| 42 | IL-6 | Peprotech | 200-06 |
| 43 | IL-7 | Peprotech | 200-07 |
| 44 | IL-21 | Peprotech | 200-21 |
| 45 | IL-22 | Peprotech | 200-22 |
| 46 | IL-23 | Peprotech | 200-23 |
| 47 | IL-31 | Prospec | CYT-625 |
| 48 | IL33 | Peprotech | 200-33 |
| 49 | IL-34 | Prospec | CYT-1069 |
| 51 | ACE2 | Sino Biological | 10108-H05H |
| 52 | CCL26 | Peprotech | 300-48 |
| 54 | CXCL10 | Peprotech | 300-12 |
| 55 | CXCL9 | Peprotech | 300-26 |
| 56 | FGF7 | Peprotech | 100-19 |
| 57 | GM-CSF | Peprotech | 300-03 |
| 58 | Leptin | Peprotech | 300-27 |
| 59 | LIF | Peprotech | 300-05 |
| 60 | LIGHT | Peprotech | 310-09B |
| 61 | MIP-1alpha | Peprotech | 300-08 |
| 62 | OPN | Peprotech | 120-35 |
| 63 | Orexin | MyBioSource | 161400 |
| 64 | Platelet derived growth factor-BB (PDGFBB) | Peprotech | 100-14B |
| 65 | sIL2R | Peprotech | 200-02RC |
| 66 | s-rank ligand | Peprotech | 310-01C |

| | | | |
|---|---|---|---|
| 67 | TMEM149 | Prospec | PRO-1432 |
| 68 | TNF-alpha | Peprotech | 300-01A |
| 69 | VEGFA | Peprotech | 100-20A |
| **Traditional Autoimmune-Associated Antigens** | | | |
| 70 | PMScl-75 | Surmodics | A17001 |
| 76 | C1q | Biodesign | A90150H |
| 78 | Bactericidal/permeability-increasing protein (BPI) | Sigma | SRP6307 |
| 79 | MDA5 | Surmodics | A30001 |
| 80 | Thyroglobulin (TG) | Surmodics | A12201 |
| 81 | Proteinase 3 | Surmodics | A18601 |
| 82 | CENPA | Surmodics | A16901 |
| 88 | Fibrillarin (FBL) | Prospec | ENZ-566 |
| 89 | Scl 70 | Surmodics | A12401 |
| 94 | Islet antigen-2 (IA2) | Novus | H00005798-Q01 |
| 96 | U1-snRNP C | Surmodics | A13201 |
| 99 | U1-snRNP A | Surmodics | A13101 |
| 100 | Pyruvate dehydrogenase (PDC-E2) | Surmodics | A17901 |
| 125 | EJ synthetase | Surmodics | A11101 |
| 127 | Thyroid peroxidase (TPO) | Surmodics | A12101 |
| 204 | Dense fine speckles-70 (DFS70) | Diarect | A30300 |
| 228 | Ro52 | Diarect | A12701 |
| 235 | Ro60/SSA | Diarect | A17401 |
| 284 | La/SSB | Diarect | A12801 |
| 285 | Sm/RNP | Immunovision | SRC-3000 |
| 309 | Smith | Immunovision | SMA-3000 |
| **Viral Antigens** | | | |
| 37 | EBV Early Antigen | Prospec | EBV-272 |

| | | | |
|---|---|---|---|
| 40 | SARS-CoV-2 Receptor binding domain (RBD) | Taia Wang | Stanford University, Stanford, CA |
| 41 | SARS-CoV-2 Nucleocapsid | | |
| 50 | EBV-EBNA1 | Prospec | EBV-276 |
| 71 | EBV Early Antigen-D | MyBioSource | MBS319448 |
| 77 | CMV glycoprotein-B | Prospec | CMV-211 |
| 84 | Influenza A hemagglutinin | Abcam | ab124596 |
| 91 | HBSAg | My BioSource | MBS142509 |

**Supplementary Table 10 - Flow Cytometry Antibodies for AIM assays**

| Epitope | Clone | Catalog Number | Company | Fluorochrome | Concentration |
|---|---|---|---|---|---|
| CD19 | HIB19 | 740287 | BD Biosciences | BUV395 | 1:25 |
| CD3 | UCHT1 | 75-0038 | Tonbo Biosciences | V450 | 1:25 |
| CD14 | M5E2 | 561392 | BD Biosciences | V500 | 1:25 |
| CD38 | HIT2 | 303531 | Biolegend | BV605 | 1:25 |
| HLA-DR | G46-6 | 563696 | BD Biosciences | BV711 | 1:25 |
| CD8 | SK1 | 35-0087 | Tonbo Biosciences | FITC | 1:25 |
| CD137 | 4B4-1 | 309803 | Biolegend | PE | 1:25 |
| OX40 | Ber-ACT35 | 350012 | Biolegend | PE-Cy7 | 1:25 |
| CD4 | OKT4 | 20-0048 | Tonbo Biosciences | APC | 1:25 |
| CD69 | FN50 | 560912 | BD Biosciences | APC-Cy7 | 1:25 |
| Viability Dye | - | 50-105-2992 | Tonbo Biosciences | V510 | 1:100 |

**REFERENCES**


1. *Salaffi F, Di Carlo M, Farah S, Marotto D, Atzeni F, and Sarzi-Puttini P. Rheumatoid Arthritis disease activity assessment in routine care: performance of the most widely used composite disease activity indices and patient-reported outcome measures. Acta Biomed. 2021;92(4):e2021238.*
2. *Gladman DD, Ibañez D, and Urowitz MB. Systemic lupus erythematosus disease activity index 2000. The Journal of rheumatology. 2002;29(2):288-91.*
3. *Rosenbach M, Murrell DF, Bystryn JC, Dulay S, Dick S, Fakharzadeh S, Hall R, Korman NJ, Lin J, Okawa J, et al. Reliability and convergent validity of two outcome instruments for pemphigus. The Journal of investigative dermatology. 2009;129(10):2404-10.*
4. *Mikuls TR, Johnson SR, Fraenkel L, Arasaratnam RJ, Baden LR, Bermas BL, Chatham W, Cohen S, Costenbader K, Gravallese EM, et al. American College of Rheumatology Guidance for the Management of Rheumatic Disease in Adult Patients During the COVID-19 Pandemic: Version 3. Arthritis Rheumatol. 2021;73(2):e1-e12.*
